# Multilevel superposition for deciphering the conformational variability of protein ensembles

**DOI:** 10.1093/bib/bbae137

**Published:** 2024-03-31

**Authors:** Takashi Amisaki

**Affiliations:** Department of Biological Regulation, Faculty of Medicine, Tottori University, Yonago, Tottori 683-8503, Japan

**Keywords:** random effects model, structural superposition, EM algorithm, covariance matrix, principal component analysis

## Abstract

The dynamics and variability of protein conformations are directly linked to their functions. Many comparative studies of X-ray protein structures have been conducted to elucidate the relevant conformational changes, dynamics and heterogeneity. The rapid increase in the number of experimentally determined structures has made comparison an effective tool for investigating protein structures. For example, it is now possible to compare structural ensembles formed by enzyme species, variants or the type of ligands bound to them. In this study, the author developed a multilevel model for estimating two covariance matrices that represent inter- and intra-ensemble variability in the Cartesian coordinate space. Principal component analysis using the two estimated covariance matrices identified the inter-/intra-enzyme variabilities, which seemed to be important for the enzyme functions, with the illustrative examples of cytochrome P450 family 2 enzymes and class A $\beta$-lactamases. In P450, in which each enzyme has its own active site of a distinct size, an active-site motion shared universally between the enzymes was captured as the first principal mode of the intra-enzyme covariance matrix. In this case, the method was useful for understanding the conformational variability after adjusting for the differences between enzyme sizes. The developed method is advantageous in small ensemble-size problems and hence promising for use in comparative studies on experimentally determined structures where ensemble sizes are smaller than those generated, for example, by molecular dynamics simulations.

## INTRODUCTION

Based on the structures deposited in the Protein Data Bank (PDB), more than 200 000 protein structures have been determined experimentally, and their number is further increasing. In this context, it is important to develop an effective method for drawing connections between functions and structures.

A protein’s function directly depends on its static structures as well as on its conformational dynamics and variability [1–5]. The covariance matrix of atomic coordinates is a traditional representation of variability. Because of its high dimensionality, it is difficult to accurately determine the off-diagonal components. Fortunately, most protein motions are considered to occur in a subspace spanned by only a small number of eigenvectors of the covariance matrix, which is referred to as essential dynamics [6, 7]. In this case, principal component analysis (PCA) has been used extensively to understand the dynamic nature of protein conformations, primarily in the analysis of trajectories generated by molecular dynamics (MD) simulations [6, 8, 9]. PCA is also applicable to ensembles composed of a small number of X-ray structures [10–15], owing to its dimensional reduction feature. Conformational variability and dynamics have been elucidated from the sample covariance matrices of X-ray structures using PCA.

In some situations, a certain ensemble hierarchy can be observed in the structural data. For example, the X-ray structure of a protein may display different conformations depending on the type of ligand bound to it. In this case, trivial grouping is used to form ensembles based on the ligand type. The variability between ensembles should indicate how the ligands affect the protein’s conformational heterogeneity. Other factors that affect conformational differences, such as crystallization conditions or sequence variants, are expected to be balanced between the ensembles; hence, the ligand effect can be efficiently estimated. Thus, introducing a two-level hierarchy by partitioning the data into ensembles by a factor is one way to distinguish factor-specific variability from variations arising from other factors. Similar ideas have been proposed in reports on simulation data [16, 17].

The factor-specific and remaining variabilities are referred to as the inter- and intra-ensemble variabilities, respectively. We can consider the latter to refer to factor-adjusted variability rather than variability arising entirely from noise. This is an important aspect of the multilevel approach. For example, when an active-site motion essential for function is shared between family enzymes that differ in the sizes of their active sites, the structural variability of the active site should be observed as a mixture of those arising from the motion and difference in sizes. By removing the inter-ensemble variability caused by the variation in size between ensembles (enzymes), the remaining variability could be the factor (size)-adjusted variability originating from the universal motion of the specific enzyme family.

Factor-based and factor-adjusting approaches to variability can be realized using a random effects model (REM) [18], which is a statistical multilevel model or mixed-effects model without regression coefficients [19] and is composed of a mean and two random variables that represent inter- and intra-ensemble deviations from the mean.

The purpose of this study was to develop a method based on the REM to estimate the covariance matrices in Cartesian coordinate space and demonstrate that the estimates are sufficiently accurate for PCA studies on currently available X-ray structures. First, the method was established by taking additional account of heterogeneous and anisotropic properties of variances. Next, using machine-synthesized data, the accuracy of the estimated covariance matrices was compared with those of alternative methods, particularly for dependencies on the ensemble size and number. Subsequently, the method was applied to the two practical datasets of X-ray structures. One is the application to the enzymes of cytochrome P450 family 2 (CYP2), which is an illustrative example of the factor-adjusting approach, and the other is an example of enzymes of class A $\beta$-lactamases (ABLs). The usefulness of this method was demonstrated using these examples. Similar utility can be expected to be found in further comparative studies on protein structures.

## MATERIALS AND METHODS

### Multilevel superposition and variance–covariance estimation

Multilevel superposition is a structural superposition built on an REM. More details on this theory are provided in Supplementary Section S1.

#### Statistical models and assumptions

Suppose that there are $\nu$ ensembles and that ensemble $i$ is composed of ${m}_i$ structures, ${\left\{{X}_{ij}\right\}}_{j=1,\cdots, {m}_i}$. An $n\times 3$ matrix ${X}_{ij}$ contains the Cartesian coordinates of $n$ atoms in three-dimensional space. Let ${Y}_{ij}$ be the coordinates obtained by translating and rotating ${X}_{ij}$,


(1) \begin{equation*} {Y}_{ij}=\left({X}_{ij}-{1}_n\otimes{t}_{ij}^T\right)\;{R}_{ij}, \end{equation*}

where $\otimes$ and ${\kern0em }^T$ denote the Kronecker product and transpose, respectively, ${t}_{ij}$ is the $3\times 1$ vector, ${R}_{ij}$ is the $3\times 3$ rotation matrix and ${1}_n$ represents the size $n$ vector of ones. In this study, the following hierarchical model was assumed for ${Y}_{ij}$:


(2) \begin{equation*} {Y}_{ij}=M+{Z}_i+{E}_{ij}\kern1em \left(j=1,\cdots, {m}_i,\kern1em i=1,\cdots, \nu \right), \end{equation*}

where $M$ is the population mean conformation and ${Z}_i$ and ${E}_{ij}$ are random deviations and all are $n\times 3$ matrices. This model is also referred to as a multilevel or mixed model. Using this model, we can distinguish between inter- and intra-ensemble variabilities. ${Z}_i$ represents the deviations of the conformations in the $i$-th ensemble from $M$ and is assumed to be independent and identically distributed as follows:


(3) \begin{equation*} \mathrm{vec}\left({Z}_i^T\right)\sim N\left(0,W\right)\kern1em \left(i=1,\cdots, \nu \right), \end{equation*}

where $W$ is the $3n\times 3n$ covariance matrix of anisotropic inter-ensemble variability. The operator $\mathrm{vec}$ represents vectorization, and for an $n\times 3$ matrix $A$, the corresponding vector $a$ was defined as $a=\mathrm{vec}\left({A}^T\right)$ in this study. In contrast, ${E}_{ij}$ is a deviation unique to the conformation ${Y}_{ij}$. All ${E}_{ij}$s were assumed to be independent and identically distributed as follows:


(4) \begin{equation*} \mathrm{vec}\left({E}_{ij}^T\right)\sim N\left(0,\varSigma \right)\kern1em \left(j=1,\cdots, {m}_i,\kern1em i=1,\cdots, \nu \right). \end{equation*}

The covariance matrix $\varSigma$ represents anisotropic intra-ensemble variability. In mixed models for clustered data ([19], pp. 2–4), ${E}_{ij}$ in Equation (2) is usually considered as an error term that is not accounted for by the ensemble deviation ${Z}_i$. When the estimation of the error variance is not the main objective, the assumption of identical distribution is usually introduced and an effect on the sample size is expected, that is, the effective sample size increases when compared to the case where the individual covariance matrix ${\varSigma}_i$ is used for each ensemble. In addition, in this study, the assumption also presents another benefit; the matrix $\varSigma$ contains the information on the conformational variability that all the ensembles have in common, owing to the assumption of identical distribution.

Notice the heterogeneity of $\varSigma$, which is the heterogeneity among the variances concerning the atoms, i.e. the diagonal components of $\varSigma$. In this paper, this is referred to as heteroscedasticity and is important in the structural superposition, as will be shown later.

#### Method of REM

The multilevel model presented in Equation (2) is a special case of mixed models, that is, the REM. In this study, a method was developed for estimating covariance matrices under the REM, and this method is referred to as the REM. In this method, the distributions of whole ensembles are considered in the superposition of individual ensembles. This procedure is based on the framework of Laird and Ware [18], in which the expectation–maximization (EM) algorithm for the maximum likelihood approach is used. The EM algorithm was used to maximize the likelihood function given as Supplementary Eq. (S7), with respect to the population parameters $M$, $W$ and $\varSigma$. The procedure is guaranteed to converge to a stationary point, i.e. the maximum likelihood estimators (Supplementary Section S1.2). One remarkable aspect of the EM algorithm is its use of unobserved latent variables, such as ${Z}_i$ in this study, as if they were observed. Consequently, in this study, the tentative estimate for the ensemble mean structure $M+{Z}_i$ was used as a target for superposition at each step of the EM algorithm, enabling the application of the REM to the superposition problem. Moreover, the estimation of ${Z}_i$ followed an empirical Bayes approach, where the tentative estimates of $W$ and $\varSigma$ were integrated into a prior distribution. The incorporation of this approach is anticipated to improve the reliability of the mean ensemble structure $M+{Z}_i$, particularly for small ensembles (with low ${m}_i$).

#### Iterative weighted least squares

In this study, for a single ensemble, the iterative weighted least squares (IWLS) [20, 21] method modified for superposition was used to estimate the covariance matrix. Similar methods were reported by Theobald and Wuttke [22]. In these methods, by taking care of the variance heterogeneity among atoms, i.e. heteroscedasticity, less fluctuating regions are tightly superimposed leaving the terminals, for example, fluctuating significantly. Under the assumption of heteroscedastic errors among atoms, as compared to ordinary least squares (OLS), which assumes homoscedastic errors, superposition is expected to be more successfully achieved using these methods in the sense that the estimators concerning superposition are statistically efficient. Although the IWLS in this study does not necessarily converge, convergence is usual [23]. A rough estimate of its computational cost was expected to be proportional to the ensemble size.

#### Two-stage method

For multi-ensemble data, to estimate the covariance matrices $W$ and $\varSigma$, the IWLS method can be used in two stages (TSs): first for each ensemble, then for the group of ensemble mean structures. Specifically, the ensemble mean structure $M+{Z}_i$ of each ensemble $i$ is first estimated using IWLS. Next, the $M+{Z}_i$ estimates of all ensembles are superimposed to obtain $M$ and $W$. $\varSigma$ is estimated as a pooled variance using the covariance matrices estimated at the first stage (Supplementary Section S1.3). This TS strategy is referred to as TS or TS/IWLS. This is another approach for the multilevel model in Equation (2), in which ${Z}_i$ are considered as population parameters with fixed values rather than random variables (that is, the fixed effects model).

#### Single pooled ensemble method

As a reference for the comparison of multilevel methods, multi-ensemble data were merged into a single pooled ensemble (SPE), on which the IWLS estimation was performed assuming


(5) \begin{equation*} {Y}_{ij}=M+{E}_{ij}\ \mathrm{and}\ \mathrm{vec}\left({E}_{ij}^T\right)\sim N\left(0,\varDelta \right)\kern1em \left(j=1,\cdots, {m}_i,\kern1em i=1,\cdots, \nu \right). \end{equation*}

The population parameters $M$ and $\varDelta$ are estimated. This conventional method is referred to as the SPE method or SPE/IWLS. Note the relation $\varDelta \approx W+\varSigma$.

### Numerical tests on synthetic data

Numerical tests were carried out on machine-synthesized data to investigate the effects of the number of ensemble $\nu$ and ensemble sizes ${m}_i$ on the accuracy of REM and TS methods. The generated conformations were those of a nucleotide pool–sanitizing enzyme, hMTH1, with $n=156$ C$\alpha$ atoms. Details of the numerical tests are described in Supplementary Section S2. Briefly, the mean conformation $M+{Z}_i$ of the $i$-th ensemble was randomly generated from a multivariate normal distribution with mean $\mathrm{vec}\ \left({M}^T\right)$ and variance–covariance $W$, which was then used, along with the variance–covariance $\varSigma$, to generate conformations belonging to that ensemble. Trajectory data obtained from a series of MD simulations on hMTH1 were used to obtain the TS estimates of $M,W$ and $\varSigma$, which were used as true matrices for the numerical tests. Accuracy of the estimated variance $\hat{v}$ was assessed in terms of the mean absolute error (MAE) over 20 replicates. Accuracy of the eigenvectors was assessed based on their overlap with the corresponding true vectors.

### Application to X-ray structures

#### Human CYP2 enzymes

The following six human enzymes in the CYP2 family were chosen, with the codes in parentheses representing their UniProt IDs:

2A6 (P11509), 2B6 (P20813), 2C8 (P10632), 2C9 (P11712), 2D6 (P10635) and 2E1 (P05181).

All PDB files listed in the structures section of the UniProt pages were downloaded. The amino acid sequences were aligned using MAFFT [24]. Conformations (PDB chains) lacking many residues were omitted. Residues were excluded from the analysis if many conformations were missing. Consequently, a dataset composed of complete cases of 163 conformations of 407 C$\alpha$ atoms was obtained. Among them, a severe outlier (PDB code 5w0c) was excluded from the final dataset. The PDB and chain IDs of the 162 conformations are listed in Supplementary Table S1.

Each ensemble was constituted by the structures of an identical enzyme, and covariance matrices $W$ and $\varSigma$ were estimated using REM and were subjected to the eigen-decomposition using the function eigen in R. For comparison, using SPE/IWLS, the covariance matrix $\varDelta$ for the pooled ensemble members was estimated and similarly factorized.

#### ABLs

The following 14 enzymes of ABLs were chosen, with the codes in parentheses representing their UniProt IDs:

BEL-1 (Q3SAW3), BPS-1 (H7C785), cTEM-19 m (P16897), CTX-M-14 (Q9L5C7), CumA (P52664), GES-1 (Q9KJY7), KPC-2 (Q9F663), L2 (Q9RBQ1), NmcA (P52663), PenL (Q2T5A3), PSE-4 (P16897), SHV-1 (P0AD64), SME-1 (Q54488) and TEM-1 (P62593).

All PDB files identified by the UniProt IDs on the PDB site were downloaded, except for CTX-M-14, KPC-2, SHV-1 and TEM-1, for which the top 25 matched files were downloaded. Although the majority of files adopted Ambler numbering [25], certain inconsistencies were found for some enzymes. Thus, the sequences were first aligned and renumbered for each enzyme, after which the sequences of all enzymes were aligned using MAFFT. Incomplete chains and residues were omitted. Finally, a dataset composed of complete cases of 149 conformations of 255 C$\alpha$ atoms was obtained. The PDB and chain IDs of the conformations are listed in Supplementary Table S2. Each ensemble was constituted by the structures of an identical enzyme, and covariance estimation and eigen decomposition were performed as described for CYP2.

#### Miscellaneous software

The REM and IWLS were coded in C and compiled using GCC 12.1.0. TS was implemented as a bash script in which IWLS and R 4.2.1 [26] were used. R was also used to evaluate accuracy. Molecular drawings were prepared using VMD 1.9.3 [27].

## RESULTS AND DISCUSSION

### Numerical tests on the synthetic data

#### Estimates of variances

**Figure 1:**
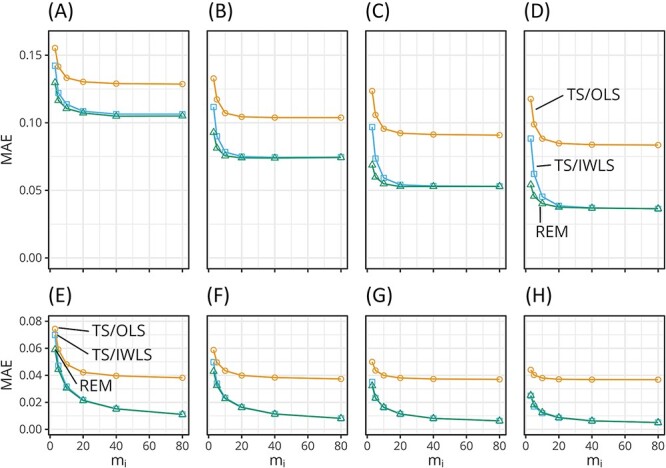
The MAE of the estimates of standard deviations. The upper four panes are the plots of the MAE of the square root of the diagonal components of $\hat{W}$ for $\nu =$ 10 (**A**), 20 (**B**), 40 (**C**) and 80 (**D**). The lower panes are the respective plots for $\hat{\varSigma}$. The averages for true values for $W$ and $\varSigma$ were 0.52 and 0.51, respectively. Lower MAE indicates higher accuracy. The accuracy of the TS/OLS estimates was lower than those of the other two methods. The accuracy of the REM $\hat{W}$ was slightly higher than the other two at low ${m}_i$.

The results of the accuracy of the diagonal elements of anisotropic covariance matrices, that is variances, are shown in Figure 1.

In Figure 1, the MAEs of the square root of variances in $\hat{W}$ and $\hat{\varSigma}$ are plotted as functions of the ensemble size ${m}_i$. Each value represents the average of over 20 replicates and over $156\times 3=468$ diagonal components.

The value of MAE decreased with increasing ${m}_i$ and with increasing $\nu$ for every method. The MAEs of the TS/OLS estimates were generally higher than those of the other estimates. The MAEs of the REM estimates of the inter-ensemble standard deviations derived from $\hat{W}$ were lower than those of TS in the region where ${m}_i$ was low, and the differences increased with the increase in $\nu$. The MAEs of the estimates of the intra-ensemble standard deviations derived from $\hat{\varSigma}$ were low compared with those with inter-ensemble variability. The larger effective sample size for estimating $\varSigma$ ($\sum_i^{\nu }{m}_i$) suppressed the magnitude of error as compared with $W$ ($\nu$). The MAEs of the TS/OLS estimates were higher than those of the other estimates, as in the case of $W$. The other two methods (REM and TS/IWLS) exhibited similar MAE values.

#### Estimates of eigenvalues and eigenvectors

The results for the eigenvalues are presented in Supplementary Section S3. In short, REM estimates were more accurate than those of TS/IWLS and TS/OLS, in particular when ${m}_i$ and $\nu$ were low, although the differences were small compared with the results for the variance estimation.

**Figure 2:**
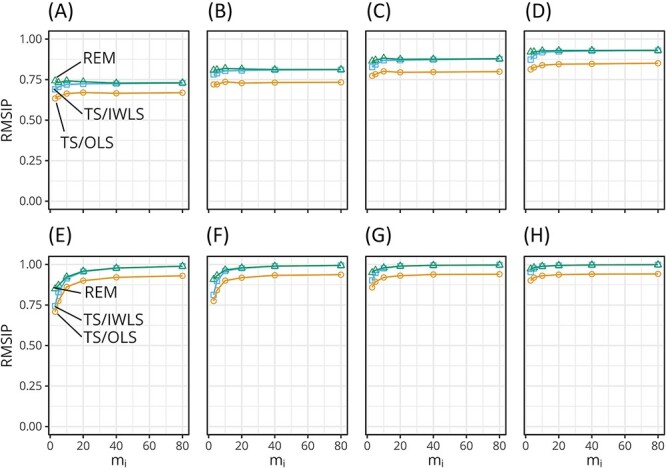
The RSMIP values of the top two eigenvectors as a function of ${m}_i$. The upper four panes are the plots for $\hat{W}$ with $\nu =$ 10 (**A**), 20 (**B**), 40 (**C**) and 80 (**D**). The lower panes are the respective plots for $\hat{\varSigma}$. At low ${m}_i$, the accuracy of REM estimates was slightly higher than those of the other methods.

The accuracy of the eigenvector estimates was evaluated in terms of the mean overlap between the space spanned by the estimates and those of the true vectors. Figure 2 shows the root mean squared inner product (RMSIP) values [28] for the top two dominant eigenvectors of $\hat{W}$ and $\hat{\varSigma}$ as functions of ${m}_i$. The RMSIP values were generally high and acceptable as concerning the top two eigenvectors. The RMSIP values for $\hat{W}$ were generally lower than those for $\hat{\varSigma}$. An increase in ${m}_i$ effectively improved the accuracy with respect to $\hat{\varSigma}$.

In general, on both $W$ and $\varSigma$, there were no large differences between the accuracy of REM and TS/IWLS, except at a low ${m}_i$ where the accuracy of REM was slightly higher than that of TS/IWLS. This difference disappeared in the results of the top eight eigenvectors (Supplementary Figure S2). The RMSIP values for the TS/OLS estimates were generally lower than those of the other two estimates at every ${m}_i$ and $\nu$. Similar results were obtained for the top eight eigenvectors.

#### Stability of estimating computation

In the numerical tests of TS/IWLS with ${m}_i=3$, the code used in this study did not properly converge to finite values at a rate of once every 14 IWLS estimations. There were no such cases concerning the other methods or other values of ${m}_i$.

### Applications to ensembles of X-ray structures

#### CYP2 family enzymes

The first example is the application to CYP2 family enzymes, which play a major role in drug metabolism, along with other families such as CYP1 and CYP3. Six major human enzymes from the CYP2 family were selected: 2A6, 2B6, 2C8, 2C9, 2D6 and 2E1.

CYP enzymes have F- and G-helices and a loop that connects them. The open/closed motion and flexibility of this F–G region are believed to be related to the uptake and release of their ligands [29, 30]. The motion of the F–G region significantly affects the cavity size of the active site [31]. In addition, the size of the active site varies between the member enzymes [32].

**Figure 3:**
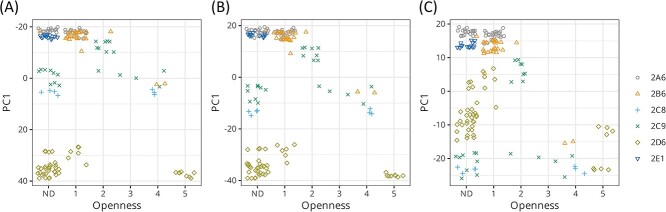
The plot of openness and coordinates of the CYP2 structures along the PC1 of the covariance matrices: (**A**) PSE/IWLS $\hat{\varDelta}$, (**B**) REM $\hat{W}$ and (**C**) REM $\hat{\varSigma}$. Openness was inferred from descriptions in the literature. ND is the abbreviation for non-determined and indicates that informative descriptions were not found in the literature. The PC1 of REM $\hat{\varSigma}$ seems to have a better correlation with openness. Notice the difference in the scale of the vertical axes.

Figure 3 shows the relationship between the openness of the structures and their coordinates along the principal component 1 (PC1) of the estimated covariance matrices. The openness of each structure was inferred from descriptions in literature. The openness score of 5 indicated that an open conformation is highly likely (PDB codes:2f9q and 3tbg). The score of 4 was assigned when the structure is likely in an open conformation, deduced from descriptions such as substantially more open (3ua5) [33], relatively open (1r9o) [34], expanded through the opening (1pq2) [35] and more open than (5xxi) [36]. The score of 3 was assigned to structures that seemed to be in intermediate states between open and closed. More closed structures were defined as those having a score of 2, and structures were defined as having a score of 1 when clearly stated as closed conformations in the references. No scores were attached to the structures without any description of open or closed status in the literature, and these structures were classified as non-determined (ND). For reference, open structures were labeled with their PDB codes in the PC1–PC2 plot of each estimated covariance matrix (Supplementary Figure S3).

The results for $\hat{W}$ and $\hat{\varDelta}$ were very similar except for the sign on PC1. On the other hand, the PC1 component in $\hat{\varSigma}$ seemed more directly related to the open/closed status than the other two. In the plot for $\hat{\varSigma}$ (Figure 3C), the open structures were located in the region where PC1 was negative, whereas the majority of closed structures indicated a positive PC1. Thus, the PC1 of $\hat{\varSigma}$ appeared to capture the variation arising from the open/close motion, which is shared between all the six enzymes. On the other hand, in the plots for $\hat{W}$ and $\hat{\varDelta}$, it was difficult to separate between the open and closed structures using the values on PC1 alone. The PC1 of $\hat{W}$ indicates a difference between the enzymes, with one possible difference being the cavity size.

To confirm this observation, the cavity dimensions were compared (Table 1). The average cavity sizes were in the order $2\mathrm{D}6>2\mathrm{C}8>2\mathrm{C}9>2\mathrm{E}1>2\mathrm{A}6\approx 2\mathrm{B}6$, which was consistent with the order observed for the values of PC1 in Figure 3A and B.

**Table 1:** Average distances showing the cavity size of each enzyme^a^. The values are the averages over the whole conformations of each enzyme

Enzyme	dist1	dist2	dist3
2A6	9.3	10.7	14.4
2B6	10.4	9.1	14.9
2C8	12.9	12.1	17.4
2C9	12.2	10.5	17.3
2D6	12.8	12.3	24.9
2E1	9.8	10.1	16.1

^a^Dist1 is the distance between two C$\alpha$ atoms of residues 50 and 213. Dist2 is the distance between the centroid of C$\alpha$ atoms of residues 103–106 and 108 and that of 232–235. Dist 3 is the distance between the centroid of C$\alpha$ atoms of residues 103–106 and 108 and that of 476–478. Dist2 and dist3 were defined in accordance with Ref. [36].

**Figure 4:**
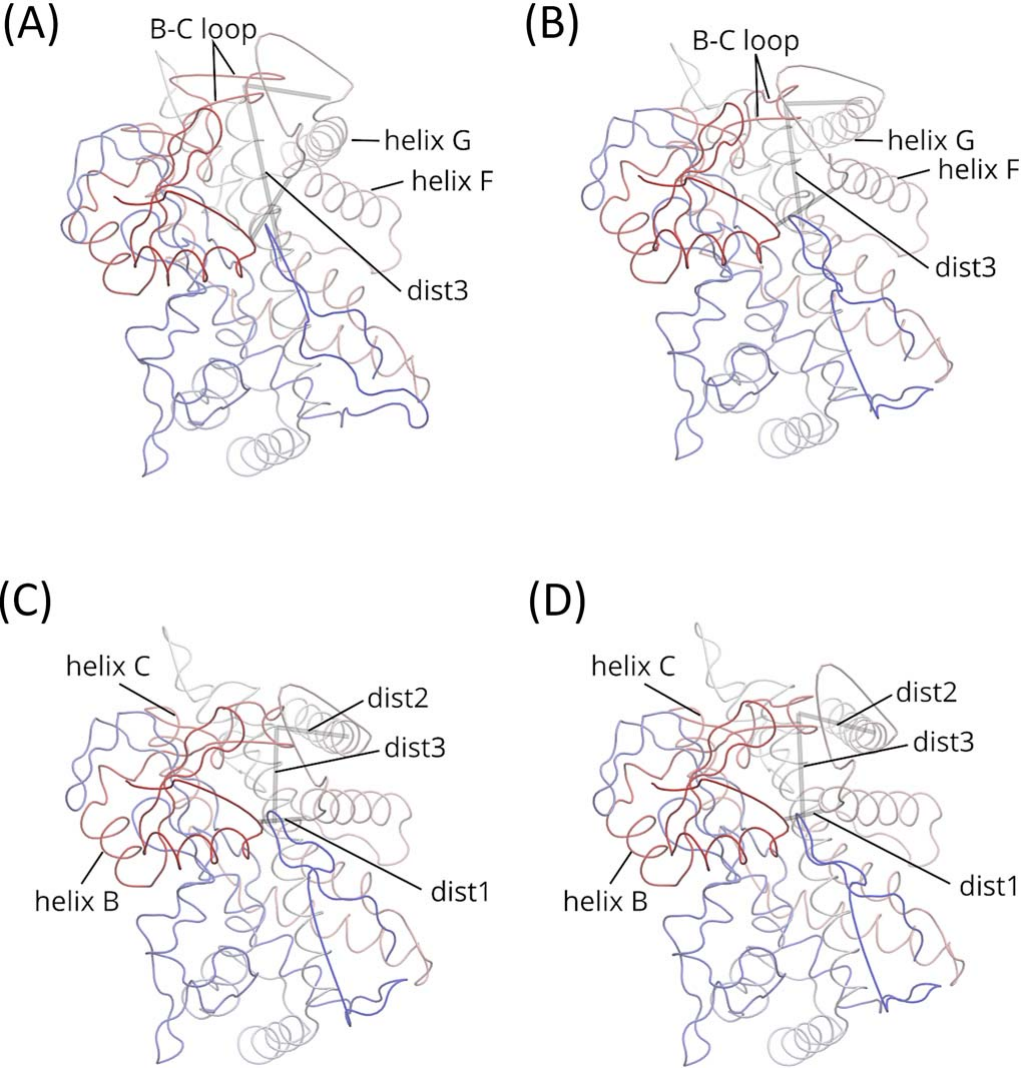
Open and closed structures of CYP2 generated in accordance with the PC1 of the estimated covariance matrices. The traces of C$\alpha$ are colored by the residue index, from red (C-terminal) to blue (N-terminal). The open structures (**A**) and (**B**) were obtained by adding the highest coordinates of the PC1 of SPE $\hat{\varDelta}$ and REM $\hat{\varSigma}$, respectively, throughout the structures to the estimated mean structures. The closed structures (**C**) and (**D**) were obtained by similarly adding the lowest coordinates of the PC1 of SPE $\hat{\varDelta}$ and REM $\hat{\varSigma}$, respectively. Structures (A) and (B) show the larger cleft produced by the upward shift of the F–G region. In addition, in structure (A), the upward shift of the B–C loop is remarkable, enlarging the active site cavity as shown by the elongated rod dist3. The three rods are drawn as defined in Table 1.

I then investigated what the PC1 coordinate corresponded to in the Cartesian coordinate space. The coordinates in PC1 of $\hat{\varDelta}$ and $\hat{\varSigma}$ were projected onto the Cartesian coordinate space and then added to the estimated average structures $\hat{M}$. Figure 4A and C shows the structures corresponding to the conformations with the maximum and minimum PC1 values for $\hat{\varDelta}$, respectively. Similarly, Figure 4B and D shows the respective structures for $\hat{\varSigma}$. The three rods in each drawing show the dimensions of the cavity, as defined by dist1, dist2 and dist3 (Table 1). In transitions (C) to (A) and (D) to (B), the F–G region (helices F and G with a loop connecting them) shifted upward and outward to enlarge the substrate-binding cleft, as indicated by the elongated rod dist1. Such a change in the location of the F–G region is likely related to the transition between the open and closed conformations. It should be noted that no significant difference was observed between the two ‘closed’ structures (C) and (D). On the other hand, the two ‘open’ structures revealed remarkable differences in the location of the B–C loop. In structure (A), the B–C loop was shifted upward, and thus, the cavity was enlarged compared to that in structure (B), as indicated by the length of the rod dist3. The results implied that the PC1 of REM $\hat{\varSigma}$ captured the open/close motion of the active site cavity, whereas the PC1 of SPE $\hat{\varDelta}$ and REM $\hat{W}$ incorporated the motion as well as the difference in the cavity sizes of the enzymes. Transitions (A)–(C) and (B)–(D) are also shown in the movies in Supplementary Section S4.

**Figure 5:**
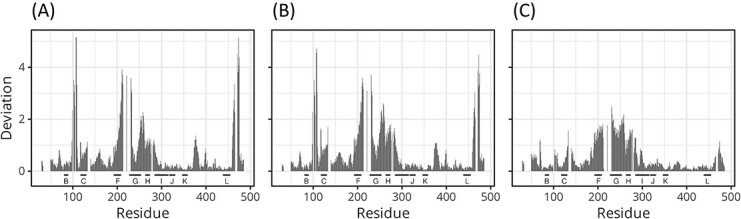
The average magnitudes of per-residue deviations of the CYP2 conformations along PC1. (**A**) SPE $\hat{\varDelta}$, (**B**) REM $\hat{W}$ and (**C**) REM $\hat{\varSigma}$. Each deviation is the average over the 162 conformations. The residue numbers of 2C8 are shown. The capital letters and bars at the bottom indicate the major helices and their regions (adopted from Ref. 37).

Figure 5 shows the per residue displacements of PC1 in the Cartesian coordinates space averaged over the 162 conformations. The values are the root mean squared deviations over 162 structures in the $x,y,z$ directions. The PC1 of REM $\hat{\varSigma}$ mainly captured the displacements around the F–G region (192–254), whereas the PC1 of REM $\hat{W}$ and SPE/IWLS $\hat{\varDelta}$ were composed of the motion of the F–G region as well as the displacements of the B–C loop (102–107) and of the C-terminal region (464–475). Spatial locations of the heavily deviated regions are shown in Supplementary Figure S4.

In summary, the results suggested that the REM estimated covariance matrix $\hat{\varSigma}$ contained the variability arising from the open/close motion of the CYP2 family after being adjusted for the differences in the native cavity sizes of the member enzymes.

#### ABLs

The second example is the application of ABLs. ABLs are active-site serine enzymes, similar to class C and D enzymes, and have been intensively studied [37]. The prevalence of carbapenemases and extended spectrum beta lactamases (ESBLs), many of which are members of class A (ABLs), is a great concern to public health. ABLs share a similar overall structural architecture surrounding the active site [38, 39]. The mobility of their loops is reportedly related to ABL function [40]. One of the important loops is the $\varOmega$-loop, which is a ‘hot spot’ for substitutions that extend the substrate spectrum of ABLs [41, 42].

**Figure 6:**
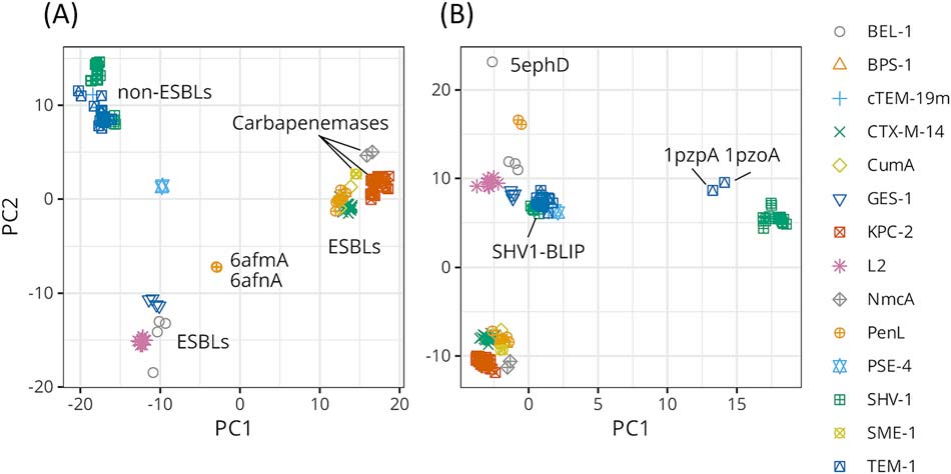
Projections of the X-ray structures of the $\beta$-lactamases onto the PC1–PC2 plane of the covariance matrices: (**A**) REM $\hat{W}$ and (**B**) REM $\hat{\varSigma}$. (A) The structures were classified into loosely three clusters: non-ESBLs, half of the ESBLs, and carbapenemases and the other half of the ESBLs. The outliers, 6afmA and 6afnA, of PenL are the structures of a non-catalytic-region mutant C69Y [39]. (B) The outliers of SHV-1 and TEM-1 were neighbors of each other’s major clusters. The structures 1pzpA and 1pzoA were reported as core-disrupted structures [43]. All structures of the SHV-1-BLIP complex were located in the vicinity of the TEM-1 cluster. The other three chains (A–C) of 5eph were not included in the analysis because of the insufficient coordinates of C$\alpha$ atoms.

Figure 6A shows the projections of the structures onto a plane spanned by the top two eigenvectors (PC1 and PC2) of the REM-estimated covariance matrix $\hat{W}$. On the PC1 coordinates, carbapenemases were on the positive extreme, whereas non-ESBLs were on the negative extreme. Half of the ESBLs were located near the carbapenemases, and the other half were in the negative region approximately halfway from the origin to the non-ESBLs.

**Figure 7:**
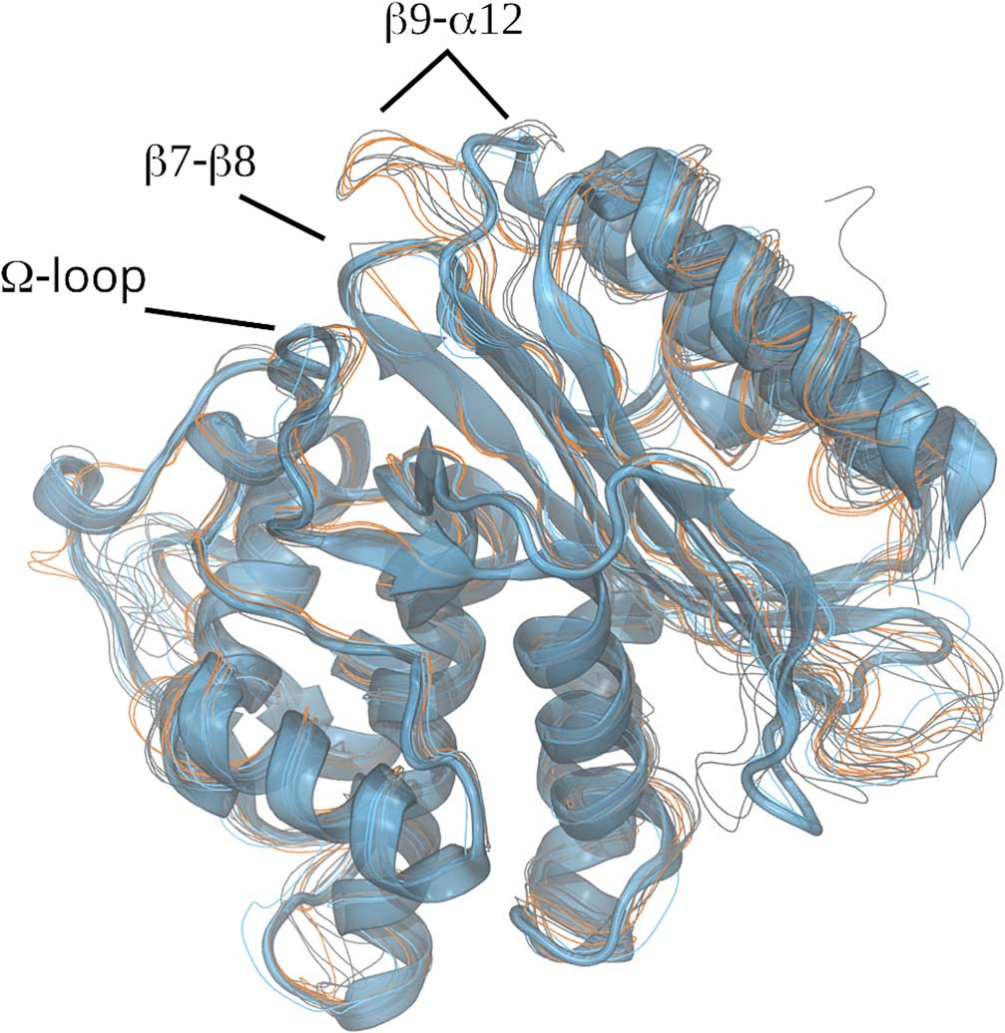
The superimposed mean structures of the $\beta$-lactamases. The cartoon-drawing is the mean structure of TEM-1. The other structures are shown in colored traces. The gray, blue and orange lines are the structures of ESBL, non-ESBL and carbapenemases, respectively. The location of $\beta 9$–$\alpha 12$ loop was noticeable. The locations of the carbapenemases and the half of the ESBLs were shifted to cover the $\beta 7$–$\beta 8$ loop which faced the $\varOmega$-loop.

**Figure 8:**
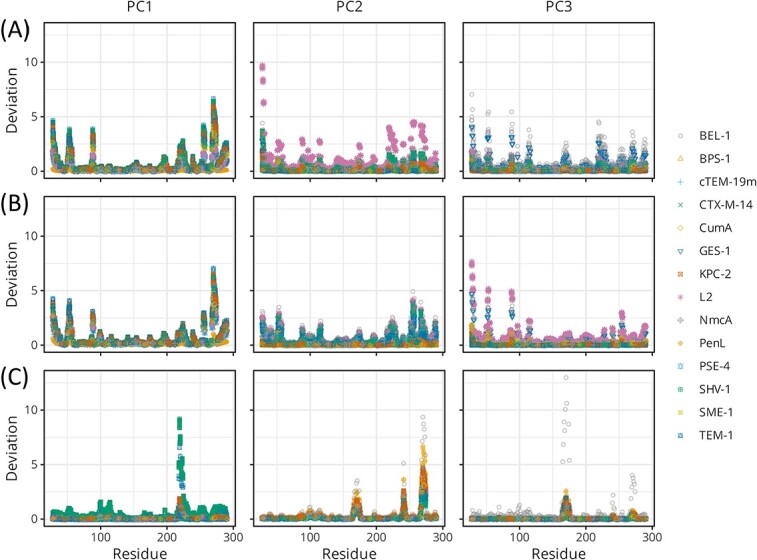
Displacement of the C$\alpha$ atoms of each structure of the $\beta$-lactamases. The coordinates in the first three PCs of (**A**) SPE $\hat{\varDelta}$, (**B**) REM $\hat{W}$ and (**C**) REM $\hat{\varSigma}$ were transformed to the deviations in the Cartesian coordinates (Å) and plotted per residue. The peak occurred at the hinge-$\alpha$11 region (218–223) for the deviation along the PC1 of $\hat{\varSigma}$ (c, left). $\varOmega$-, $\beta 7$–$\beta 8$, and $\beta 9$–$\alpha 12$ (169–176, 238–243, 266–275, respectively) constituted triple peaks in the plot for the PC2 of $\hat{\varSigma}$ (C, middle). The standalone peak in (C, right) was produced mainly by the outliers in BEL-1 (5ephD). The PC1s of $\hat{\varDelta}$ and $\hat{W}$ were almost identical as indicated by the dot product between the two as high as 0.961.

From the perspective of per-residue deviation, PC1 was comprised of peaks at several loops (Figure 8B, left), among which the highest peak in the residues 266–275 corresponded to the heterogeneity in the $\beta 9$–$\alpha 12$ region. As shown in Figure 7, this portion of the carbapenemases and half of the ESBLs covered over the edge of loop $\beta 7$–$\beta 8$ (238–243), whereas the other half of the ESBLs and non-ESBLs pulled the portion to align the edges of the $\beta 9$–$\alpha 12$ and $\beta 7$–$\beta 8$ loops and the $\varOmega$ loop C-terminal (169–176). Knoverek *et al.* reported that the penicillinase activity of TEM increases as the opening of the cryptic pocket forms between $\beta 7$–$\beta 8$ and $\varOmega$ loops [44]. Interactions between the two loops and the $\beta 9$–$\alpha 12$ loop may be related to the activity of ABLs. It is also worth noting that the change in the position of the $\beta 9$–$\alpha 12$ loop was coupled to the changes in the loops on the opposite side, i.e. $\beta 8$–$\beta 9$ (252–258) and $\beta 1$–$\beta 2$ (51–55). The conformations or motions of these portions may also be related to the activity of ABLs.

The collective motion or joint heterogeneity on the configuration of the three loops was more clearly captured in the PC2 of REM $\hat{\varSigma}$. The plot for the per-residue deviation of PC2 for REM $\hat{\varSigma}$ is composed of three peaks at the $\varOmega$, $\beta 7$–$\beta 8$ and $\beta 9$–$\alpha 12$ loops (Figure 8C, middle). As shown in Figure 6B, the PC2 values were divided into two groups. These results suggested that there are two main states for the configuration or conformation of these three loops.

Variability in the hinge conformation was detected using REM, as indicated in Figure 8C (left). Specifically, the per-residue deviation of PC1 for REM $\hat{\varSigma}$ showed a clear peak in the hinge–$\alpha 11$ region (218–223). A study using Markov state models showed that the hinge regions of TEM-1 and SHV-1 were more flexible than the highly stable hinge region of KPC-2 [45]. Other studies have reported that binding to a beta-lactamase inhibitory protein (BLIP) introduces a conformational change in the hinge region [46, 47]. These results are consistent with those shown in Figure 6B. The TEM-1 showed two distinct PC1 values depending on the ligand. The SHV1-BLIP complexes were located near the TEM-1 cluster, far from the major cluster of SHV-1.

In summary, the conformational heterogeneity captured in PC1 and PC2 of $\hat{W}$ appears to be related to the resistance profiles of the ABLs. With regard to $\hat{\varSigma}$, PC1 indicated conformational heterogeneity in the hinge region, whereas PC2 indicated joint heterogeneity at the three loops of $\varOmega$, $\beta 7$–$\beta 8$ and $\beta 9$–$\alpha 12$.

### Concluding remarks

In this study, a two-level estimation of covariance matrices in Cartesian coordinates was proposed for comparative studies of protein conformations. The method was based on the REM where variation between the ensembles of conformations ($W$) was distinguished from that within the ensembles ($\varSigma$).

One of the benefits of this method is that the $W$-adjusted variability is extracted. In the CYP2 example, active-site dynamics shared between the CYP2 enzymes were obtained as the PC1 of $\hat{\varSigma}$ being adjusted with the differences in the cavity sizes that were, in turn, obtained as the PC1 of $\hat{W}$. In the example of ABLs, within-ensemble variability ($\varSigma$) captured the joint heterogeneity of the three adjacent loops as well as the variation in the hinge region. It would be difficult to notice conformational heterogeneity in the hinge region if the traditional non-hierarchical SPE method was employed. Nevertheless, the REM-estimated variability between the ensembles differed little from that estimated by SPE in the case of the ABL structures, as indicated by the high degree of coincidence of the two PC1s indicated.

Notably, in the REM method, the covariance matrices are estimated according to the specified grouping. If the grouping was inadequate, it would be possible to identify artifacts as inter-group variability. Thus, it is important to carefully validate the estimates; for example, by referring to those of the SPE method in which no grouping is used. If a definite grouping is unknown, any clustering method can be used to determine the grouping. For example, in additional experiments, inter-ensemble variability was well estimated based on the grouping determined by *k*-medoids using root-mean-squared-deviation values. However, the estimates for intra-ensemble variability varied significantly depending on the group sizes, making them less reliable when grouping was determined by clustering methods.

In general, TS/IWLS, the other two-level method used in this study, showed results similar to those of REM. Good agreement was observed between the essential PCs estimated by REM and those estimated by TS/IWLS in the X-ray structure results, except in a few cases. In general, REMs are more efficient (accurate) than fixed effects models (e.g. TS) in the cases where the ensemble sizes are small in comparison with the number of ensembles, ${m}_i<\nu$ [19]. In addition, the REM method in this study copes with deviations ${Z}_i$ in the empirical Bayes framework, which also implies its advantage in such cases. In other words, information from the other ensembles was used to compensate for the poor sample size ${m}_i$ when estimating ${Z}_i$ and superimposing the structures of ensemble $i$.

One drawback of REM over TS is its high computational cost. For example, the execution times of REM for CYP2 and ABL were 1117 and 240 s, respectively, using a single core of Intel(R) Core(TM) i9-9900, whereas the respective times of TS/IWLS for CYP2 and ABL were 63.2 and 27.1 s. This advantage of TS over REM is assumed to come from its divide-and-conquer nature and would be further extended when applied to data with very large ensemble sizes (${m}_i$), which is usual in the case of MD trajectory data. The slow speed of REM was partly related to the slow convergence of the EM algorithm. The use of Newton’s or similar methods might improve this drawback to some extent. Such improvement would be more valuable in higher-level models than in the two-level models. In theory, the two-level models in this study can be easily extended to higher-level models; however, it would be computationally expensive.

The assumption of normality is not always valid and there may be cases where the methods in this work pose problems. Of particular concern are small samples with outliers. In fact, in the numerical tests of this study, TS/IWLS occasionally failed at the first stage when the ensemble size was small (${m}_i=3$). However, the normal distribution may be viewed as an approximation to the original distribution and is usually used in mixed/random effects models ([19], p. 233). In addition, the effective sample sizes are enlarged in the REM and SPE methods, because the estimations are carried out using structures of all ensembles. Nevertheless, it would be important to validate results, for example, by visually inspecting the superimposed ensemble-average structures and the projections of the structures onto the planes of the estimated PCs.

This study developed methods that employ superposition in favor of intuitively appealing Cartesian coordinates, rather than internal coordinate systems. Recently, sophisticated methods for analyzing structure ensembles in the Cartesian coordinates space were reported. Hirsch and Habeck applied a Gaussian mixture model (GMM) to model the intra-ensemble variability within a single ensemble (or within a single protein chain) [48]. Although their superposition method corresponded to OLS, the heteroscedasticity was accounted for using GMM. Jamroz *et al*. [49] proposed a score named FlexScore, which is based on the estimated mean structure and covariance matrix of a single ensemble. They used the framework of Theobald and Wuttke [22] for ensemble superposition. Lindorff-Larsen and Ferkinghoff-Borg [50, 51] proposed a similarity measure between two ensembles; that is, the Kullback–Leibler divergence between the two normal distributions, which were represented by their respective mean structures and covariance matrices. These studies concerned the distribution of a single ensemble, whereas REM and TS in this paper are methods for multiple ensembles.

A possible limitation of these methods is that they arise from superpositions. There is controversy over superposition in the context of PCA and the clustering of MD trajectories, particularly when applied to extreme structural changes and perhaps to too large ensembles [52, 53]. Nevertheless, two-level superposition alleviates this drawback by dividing the entire dataset into ensembles, which also mitigates the concern that the average structure used for superposition is not a real protein conformation. In addition, the heteroscedasticity-aware strategy of REM and IWLS is expected to improve the quality of superposition over OLS, as indicated by the results of the numerical tests.

**Figure 9:**
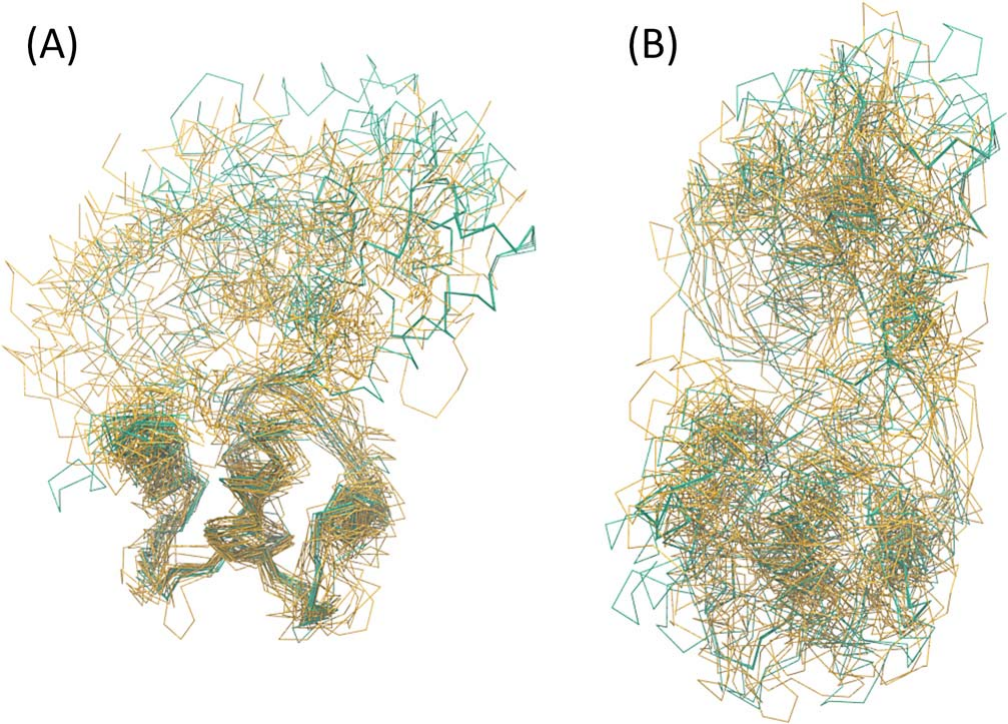
Superimposed mean group structures of calmodulin. (**A**) REM and (**B**) TS/OLS. The traces of the C$\mathrm{\alpha}$ atoms are shown. The yellow and blue lines represent the average structures of the NMR and X-ray ensembles, respectively. For example, the uppermost helix in (**A**) is the average structure of that of an X-ray ensemble. Calmodulin consists of N- and C-domains and a link connecting them. In the structures superimposed using the (heteroscedastic) REM method, compared to the C-terminal half, the N-terminal half exhibited a better fit.

In an additional application on calmodulin (Supplementary Section S5), in which a mixture of nuclear magnetic resonance (NMR) and X-ray ensembles was used, REM and IWLS successfully captured large structural changes. Calmodulin consists of two domains, N- and C-domains, that are connected by a flexible linker. The superposition (Figure 9A), obtained using the REM method, indicates that large conformational changes occurred between the two domains, which are difficult to interpret in the TS/OLS results (Figure 9B). The REM method yielded the superposition fitted solely at the N-domain despite the high structural similarity between N- and C-domains. On the other hand, when more than seven N-terminal residues were excluded from the calculation, C-domain fitted superpositions were obtained as shown in Supplementary Figure S6A. A similar result was also observed in the TS/IWLS method (Supplementary Figure S6B), although its response to the changes in the number of residues was different from that of the REM method.

It should be noted that the final superposition depends on the initial superposition, that is the problem of the multiple local minima. For the calmodulin data, some examples are shown in Supplementary Figure S7 and discussed in Supplementary Section S5 (pp. s23–s24). Thus, it may be meaningful to seek other superpositions or to confirm that the superposition is a global optimum, by initiating from different superpositions or only using subsets of data.

There seem to be several approaches for improving or extending the multilevel superposition reported in this paper. These methods are restricted to datasets of complete cases. It will be valuable to investigate methods of relaxing these restrictions, to allow for the analysis of structures with gaps and/or with disordered atoms. Another concern is that the accuracy or reliability of X-ray and NMR structures varies between experiments, unlike structures generated from MD simulations. If a measure that describes the reliability of each experiment was established, it would be used to impose differential importance on individual experiments using, for example, a composite likelihood [54], to which a major portion of the current work could be applied. Studies on the utility of the estimated covariance matrices may also yield meaningful results. For example, this information may be used in the refinement or validation of newly obtained structures. It is also interesting to incorporate a kernel approach to cope with non-linear motion and heterogeneity [55]. The superposition-free application of mixed/random effects models to the internal coordinates, e.g. inter-atomic distances and dihedral angles, seems to be straightforward, although application-specific investigations will be required. Lastly, owing to the similarity between the two methods future work incorporating GMM, as a clustering tool, in the REM method would serve as an interesting approach.

In the REM method, one of the benefits of subjecting multiple ensembles rather than a single ensemble is that the entire dataset is used to improve the superposition and estimation in small ensembles of insufficient structures. Computationally, REM is more expensive than TS on large-scale problems. These points together suggest that the REM method is well suited for problems with small-sized ensembles, particularly for experimentally obtained structures. Its results, which are given in Cartesian coordinates, may be appealing to researchers in experimental fields, although the scope of the REM method is not limited to X-ray and NMR structures. It is also interesting to apply the REM and TS methods to clusters of structures in sequence-based prediction [56]. As illustrated in this study, the REM method is useful for comparative studies of protein structures, providing a tool for investigating the dynamics and heterogeneity of protein conformations in terms of inter- and intra-ensemble variability.

**Key Points**

- A mixed-model method was developed for estimating two covariance matrices that represent inter- and intra-ensemble variabilities.
- The method incorporated multilevel superposition in the Cartesian coordinate space.
- The results showed that the conformational variation between ensembles can be effectively extracted, using principal component analysis, from the inter-ensemble covariance matrix estimated by the method proposed in this paper.
- The other covariance matrix contained information on the variability spanning the ensembles, which resulted from the removal of inter-ensemble differences from the observed variability.
- As an example of cytochrome P450 family 2 enzymes, in which each enzyme has an active site cavity of various sizes, an open/closed motion shared between the enzymes was captured in the first principal mode of the intra-enzyme covariance matrix.

## Supplementary Material

movieS1_bbae137

movieS2_bbae137

supple_bbae137

## Data Availability

Related codes and data are available at https://github.com/amisakit/mxfitci.

## Author Biographies

**Takashi Amisaki** is a professor at the Department of Biological Regulation, Faculty of Medicine, Tottori University.
