# Supplementary material for: Multilevel superposition for deciphering the conformational variability of protein ensembles
Source: Brief Bioinform. 2024 Mar 31;25(3):bbae137. doi: 10.1093/bib/bbae137 (PMC10983786; doi:10.1093/bib/bbae137)
Supplement: supple_bbae137 [file supple_bbae137.docx]

Multilevel Superposition for Deciphering the Conformational Variability of Protein Ensembles
 Supplementary Data

Takashi Amisaki${}^{*}$
*Department of Biological Regulation*
*Faculty of Medicine, Tottori University*
*Yonago, Tottori 683-8503, Japan*

# Section S1 Theoretical Details on IWLS, REM, TS, and Superposition

## S1.1 The IWLS Method

The least-squares method used in this study for a single ensemble is briefly described. The estimators were derived from the normal likelihood of the joint distribution of a single ensemble of conformations. This method corresponds approximately to that reported by Theobald and Wuttke [1], except for the working covariance matrix.

Consider only the $i$-th ensemble and rewrite Eq. (2) as

| $Y_{ij}=M_{i}+E_{ij}, \left( j=1,\cdots,m_{i} \right),$ | $\left( S1 \right)$ |
| --- | --- |

where, $M_{i}$ denotes the mean conformation. The iterative weighted least squares (IWLS) method [2,3] of this study repeats the following three steps until convergence: (i) calculate the estimate of the mean conformation $\hat{M}_{i}$, (ii) calculate the best rotational matrices and translational vectors $\left( \hat{R}_{ij},\hat{t}_{ij} \right)$, and (iii) calculate the estimate $\hat{S}$ for $S$ fixing $M=\hat{M}$ and $\left( R_{ij},t_{ij} \right)=\left( \hat{R}_{ij},\hat{t}_{ij} \right)$ for all $j$, where $S$ is the working covariance matrix, which is an $n\times n$ matrix for an isotropic approximation of $\Sigma$ defined as $\Sigma=S\otimes I_{3}$, where $I_{3}$ denotes a $3\times3$ identity matrix. The term *working* indicates that $S$ is used to estimate the other parameters. The estimators, except for $R_{ij}$, were obtained from the derivative of twice negative log-likelihood $L$ given as

| $-2L=3nm_{i} \log\left( 2\pi\right)+3m_{i} \log\left\vert S \right\vert+\sum_{j=1}^{m_{i}} \mathrm{tr}\left( Y_{ij}-M_{i} \right)^{T} S^{-1} \left( Y_{ij}-M_{i} \right)$, | $\left( S2 \right)$ |
| --- | --- |

where $\mathrm{tr}\left( A \right)$ is a trace of $A$. Note that $Y_{ij}$ is a function of the parameters $R_{ij}$ and $t_{ij}$. Steps (i) and (ii) constitute the weighted least-squares method, in which the diagonal component of $\hat{S}$, that is variance estimates, are used as inverse weights. Although the IWLS procedure does not necessarily converge, convergence is usual [4]. In this study, for comparison with the heteroscedastic weighting scheme, the traditional homoscedastic method, in which $\hat{S}$ is fixed to an identity matrix, was also used. The latter method is referred to as the ordinary least-squares (OLS) method.

The estimates $R_{ij}$ in step (ii) were calculated using the singular value decomposition of the term:

| $\mathrm{tr} M_{i} R_{ij}^{T} \left( X_{ij}-1_{n}\otimes t_{ij}^{T} \right)^{T}S^{-1},$ | $\left( S3 \right)$ |
| --- | --- |

This is the only term that depends on $R_{ij}$. This is an elegant strategy for obtaining the least-squares solution of $R_{ij}$ as proposed by Kabsch [5]. Note that this strategy can be applied in heteroscedastic cases (i.e., different weights on atoms), but not in the anisotropic form (i.e., different weights on $x,y,z$).

The anisotropic full-components $\Sigma$ was estimated as an empirical variance-covariance matrix, after the minimization of $-2L$ was attained. The estimator of $\Sigma$ was derived from the anisotropic counterpart of Eq. (S2).

## S1.2 The REM Method

The multi-level model in Eq. (2) is a random-effects model if $Z_{i}$ is considered as a matrix of random variables, as assumed in Eq. (3). In this study, the REM method was developed for estimating the covariance matrices under the random effects model. This method is based on the framework of Laird and Ware [6], in which the EM algorithm for the maximum likelihood approach, combined with empirical Bayes estimates, is used. This strategy is briefly described below.

The EM algorithm alternates between the E-step and M-step until convergence [7]. In the E-step, the expectation of complete likelihood, the $Q$-function, is prepared. In this study, the complete log-likelihood is the logarithm of the joint density of $\{Y_{ij}\}$ and $\{Z_{i}\}$, and the $Q$-function can be written using the empirical Bayes estimates $\hat{Z}_{i}$ for $Z_{i}$, as

| $\begin{matrix} & -2Q\left( M,V,S,R,t \right) \\ & =3mn\log\left( 2\pi\right)+3m\log\left\vert S \right\vert+\sum_{i} \sum_{j} \mathrm{tr}\left( Y_{ij}-M-\hat{Z}_{i} \right)^{T} S^{-1} \left( Y_{ij}-M-\hat{Z}_{i} \right) \\ & +3n\nu\log\left( 2\pi\right)+3\nu\log\left\vert V \right\vert+\sum_{i} 3m_{i} \mathrm{tr}\hat{U}_{i} S^{-1}+\sum_{i} \mathrm{tr}\left( 3\hat{U}_{i}+\hat{Z}_{i} \hat{Z}_{i}^{T} \right) V^{-1}, \end{matrix}$ | $\left( S4 \right)$ |
| --- | --- |

where $R$ and $t$ are the abbreviations for $\{R_{ij}\}$ and $\{t_{ij}\}$, respectively. Note that $\hat{Y}_{ij}$ is a function of the parameter estimates $\hat{R}_{ij}$ and $\hat{t}_{ij}$. The anisotropic covariance matrices $W$ and $\Sigma$ are respectively approximated with $V\otimes I_{3}$ and $S\otimes I_{3}$, where $V$ and $S$ are the isotropic covariance matrices for inter- and intra-ensemble variability. Matrices $\hat{U}_{i}$ and $\hat{Z}_{i}$ are calculated as follows:

| $\hat{U}_{i}=\left( m_{i} \hat{S}^{-1}+\hat{V}^{-1} \right)^{-1}, \hat{Z}_{i}=\hat{U}_{i} \hat{S}^{-1}\sum_{j=1}^{m_{i}} \left( \hat{Y}_{ij}-\hat{M} \right),$ | $\left( S5 \right)$ |
| --- | --- |

where $\hat{S}$, $\hat{V}$, $\hat{M}$, and $\hat{Y}_{ij}$ are the tentative estimates of $S,V,M$, and $Y_{ij}$, respectively. $\hat{Z}_{i}$ and $\hat{U}_{i}$ are the mean and the variance of the posterior distribution of $Z_{i}$, given $\{Y_{ij}\}$, $M$, $V$, and $\Sigma$. This Bayesian strategy is expected to improve estimation quality when $m_{i}$ is low.

The EM algorithm in this study repeats the estimations (i)–(iii) until convergence: (i) $\hat{M}$, (ii) $\hat{R}_{ij},\hat{t}_{ij}$, and (iii) $\hat{V},\hat{S}$. The estimators for these quantities, except for $\hat{R}_{ij}$ were obtained from the estimating equations which were derived by differentiating Eq. (S4). The estimates for $R_{ij}$ were obtained by the singular value decomposition (SVD) of the product of the matrices:

| $\begin{matrix} \left( \hat{M}+\hat{Z}_{i} \right) \left( X_{ij}-1_{n}\otimes g_{ij}^{T} \right)^{T} \hat{S}^{-1}, \end{matrix}$ | $\left( S6 \right)$ |
| --- | --- |

where $g_{ij}=X_{ij}^{T} \hat{S} 1_{n}/1_{n}^{T} \hat{S}^{-1} 1_{n}$, the product is the $S^{-1}$-weighted covariance between the estimated ensemble average $\hat{M}+\hat{Z}_{i}$ and conformation $X_{ij}$. This strategy is called the Kabsch method for the least-squares solution $\hat{R}_{ij}$ [5].

An EM-step never decreases the incomplete likelihood $L_{o}$, hence REM is guaranteed to converge to a stationary point, i.e., the maximum likelihood estimators. The incomplete likelihood, also referred to as the marginal likelihood, can be written as:

$$\begin{aligned} \begin{matrix} & -2\log L_{o}\left( M,V,S \right) \\ & =3mn\log\left( 2\pi\right)+3\left( m-r \right)\log\left| S \right|+\sum_{i=1}^{r} \left[ 3\log\left| S+m_{i} V \right| \right. \\ & +\sum_{j=1}^{m_{i}} \mathrm{tr}\left( Y_{ij}-M \right)^{T} S^{-1} \left( Y_{ij}-M \right) \\ & \left. -tr\Sigma^{-1} V \left( S+m_{i}V \right)^{-1}\sum_{j=1}^{m_{i}} \left( Y_{ij}-M \right) \sum_{j=1}^{m_{i}} \left( Y_{ij}-M \right)^{T} \right]. \end{matrix}\#\left( S7 \right) \end{aligned}$$

With regard to the anisotropic $W$ and $\Sigma$, an anisotropic $Q$ function, which is a counterpart for Eq. (S4) can be differentiated to derive the estimated equations. However, the estimates for $R_{ij}$ can no longer be obtained in the anisotropic case using the SVD of Eq. (S6), as in the case of IWLS. Thus in this study, after the isotropic algorithm converged, the final anisotropic full-matrix $\hat{W}$ and $\hat{\Sigma}$, the estimates for $W$ and $\Sigma$, respectively, were calculated using the isotropic estimates $\hat{U}_{i}$ as well as using the estimates $\hat{Y}_{ij}$, $\hat{M}$, and $\hat{Z}_{i}$.

The expressions of the estimators are presented in the remainder of this subsection. The tentative estimator of $M$ in an EM iteration was expressed as

| $\begin{matrix} \hat{M}=\frac{1}{m} \sum_{i=1}^{r} \sum_{j=1}^{m_{i}} \hat{Y}_{ij}-\frac{1}{m} \sum_{i=1}^{r} m_{i} \hat{Z}_{i}, \end{matrix}$ |  |
| --- | --- |

where $m=\sum_{i}^{\nu} m_{i}$. The estimate converges to the ML estimate

| $M=\left( \sum_{i=1}^{r} m_{i} \left( \Sigma+m_{i}V \right)^{-1} \right)^{-1} \sum_{i=1}^{r} \left( \Sigma+m_{i}V \right)^{-1}\sum_{j=1}^{m_{i}} Y_{ij}.$ |  |
| --- | --- |

The estimate of each rotation matrix is the product of two matrices $P Q^{T}$ which are obtained by the singular value decomposition $P \Lambda Q^{T}$ of

| $\begin{matrix} \left( X_{ij}-1_{n}\otimes g_{ij}^{T} \right)^{T} \hat{S}^{-1} \left( \hat{M}+\hat{Z}_{i} \right) \end{matrix}$ |  |
| --- | --- |

where

| $g_{ij}=\frac{1}{1_{n}^{T} \hat{S}^{-1} 1_{n}} X_{ij}^{T} \hat{S}^{-1} 1_{n}.$ |  |
| --- | --- |

The estimators of translational vectors were given as

| $\begin{matrix} \hat{t}_{ij}=g_{ij}-\frac{1}{1_{n}^{T} \hat{S}^{-1} 1_{n}} \left[ \hat{R}_{ij} \hat{M}^{T} \hat{S}^{-1} 1_{n}+\hat{R}_{ij} \hat{Z}_{i}^{T} \hat{S}^{-1} 1_{n} \right]. \end{matrix}$ |  |
| --- | --- |

The ensemble deviations were written as

| $\begin{matrix} \hat{Z}_{i} & =\hat{U}_{i} \hat{S}^{-1}\sum_{j=1}^{m_{i}} \left( \hat{Y}_{ij}-\hat{M} \right). \end{matrix}$ |  |
| --- | --- |

The isotropic covariance matrices were given as

| $\begin{matrix} \hat{V} & =\frac{1}{3r}\sum_{i=1}^{r} \left( \hat{Z}_{i} \hat{Z}_{i}^{T}+3 \hat{U}_{i} \right) \\ \hat{U}_{i} & =\hat{V} \left( \hat{S}+m_{i}\hat{V} \right)^{-1} \hat{S} \\ \hat{S} & =\frac{1}{3m} \sum_{i} \sum_{j}\left( 3\hat{U}_{i}+\left( Y_{ij}-\hat{M}-\hat{Z}_{i} \right) \left( Y_{ij}-\hat{M}-\hat{Z}_{i} \right)^{T} \right) \end{matrix}$ |  |
| --- | --- |

The off-diagonal components of $\hat{V}$, $\hat{U}_{i}$, and $\hat{S}$ were ignored in the EM iterations. The estimators of the anisotropic covariance matrices were given as follows:

| $\begin{matrix} \hat{W} & =\frac{1}{r}\sum_{i=1}^{r} \left( \hat{z}_{i} \hat{z}_{i}^{T}+\hat{U}_{i}\otimes I_{3} \right), \\ \hat{\Sigma} & =\frac{1}{m}\sum_{i=1}^{r} \sum_{j=1}^{m_{i}} \left( \left( y_{ij}-\mu-z_{i} \right) \left( y_{ij}-\mu-z_{i} \right)^{T}+U_{i}\otimes I_{3} \right), \end{matrix}$ |  |
| --- | --- |

where $y_{ij}=vecY_{ij}^{T}$, $\mu=vecM^{T}$, and $z_{i}=vecZ_{i}^{T}$. The final estimates $\hat{W}$ and $\hat{\Sigma}$ were scaled by $n/\left( n-2 \right)$. This factor corrects for the degrees-of-freedom of rotation and translation.

## S1.3 The TS method

Another way to estimate the two covariance matrices is to first calculate, for each ensemble $i$, the estimates of $M+Z_{i}$ and $\Sigma$, $\hat{Y}_{i}$ and $\hat{\Sigma}_{i}$ using the IWLS method, and then estimate the $\hat{M}$, $\hat{R}_{i}$ and $\hat{W}$ under the model $\hat{Y}_{i}=M+Z_{i}$, again using the IWLS method. The estimate for $\Sigma$ was the pooled variance-covariance calculated as

| $\hat{\Sigma}=\frac{1}{\sum_{k=1}^{\nu} m_{i}}\sum_{k=1}^{\nu} m_{i} \left( I_{n}\otimes\hat{R}_{k}^{T} \right) \hat{\Sigma}_{k} \left( I_{n}\otimes\hat{R}_{k} \right),$ | $\left( S8 \right)$ |
| --- | --- |

where $\hat{R}_{i}$ is the estimate of the rotational matrix for ensemble $i$. $I_{n}$ is an $n\times n$ identity matrix. Finally, the estimates $\hat{W}$ and $\hat{\Sigma}$ were scaled by $\nu/\left( \nu-1 \right)\cdot n/\left( n-2 \right)$ and $\sum_{k=1}^{\nu} m_{i}/\left( \sum_{k=1}^{\nu} -\nu\right)\cdot n/\left( n-2 \right)$, respectively, adjusting for the degrees of freedom.

In this strategy, $Z_{i}$ in Eq. (2) was considered as the population parameters rather than random variables, in which case Eq. (2) is a fixed-effects model. This two-stage (TS) superposition is referred to as TS/IWLS or TS/OLS if the OLS weighting scheme is used instead of the IWLS.

# Section S2 Procedures for the Numerical Tests

Trajectory data obtained from a series of molecular dynamics simulations on the nucleotide pool sanitizing enzyme hMTH1 were used to create the population mean $M$ and population variance-covariance matrices $W$ and $\Sigma$ of the coordinates of C$\alpha$ atoms ($n=156$) using TS/IWLS. As thus obtained $W$ was not positive-definite, 353 out of its 468 eigenvalues that were lower than $\epsilon_{M}^{2/3}$ were replaced with $\epsilon_{M}^{2/3}$ and the matrix was reconstructed, where $\epsilon_{M}^{2/3}\approx2.22\times{10}^{-16}$. These *true* population parameters were used to generate the conformations.

A dataset $\{X_{ij}\}$ was composed of $m_{i}\times\nu$ conformations which were drawn from the multivariate normal distribution with the mean $\mathrm{vec}\left( M^{T}+Z_{i}^{T} \right)$ and variance-covariance $\Sigma$, each for $j=1,2,\cdots,m_{i}$. An ensemble mean conformation $M+Z_{i}$ was drawn from the multivariate normal distribution with the mean $\mathrm{vec}\left( M^{T} \right)$ and variance-covariance $W$, each for $i=1,2,\cdots,\nu$. The balanced design was used, that is, $m_{1}=m_{2}=\cdots=m_{\nu}$ in a dataset. Twenty datasets were generated for each combination of $\nu=10,20,40,80$ and $m_{i}=3,5,10,20,40,80$.

The covariance matrices $W$ and $\Sigma$ were estimated using REM, TS/IWLS, and TS/OLS for these $4\times6\times20=840$ datasets. The rotation that best fits the $\hat{M}$ onto $M$ was applied to each estimated matrix. The variances, eigenvalues, and eigenvectors of each estimated covariance matrix were then calculated. The accuracy of the estimated value $\hat{v}$ was assessed in terms of the mean error of $\hat{v}-v^{*}$ (ME) and the mean absolute error of $\left| \hat{v}-v^{*} \right|$ (MAE) over 20 replications, where $v^{*}$ is the corresponding true value. The accuracy of the eigenvectors was assessed based on their mean overlap with the corresponding true vectors using the root mean squared inner product (RMSIP) [8] between the estimated and true vectors.

Occasionally, the TS/IWLS method did not converge to finite estimates in the first stage of $m_{i}=3$ dataset. In such cases, the estimates in the second stage were calculated using only the successfully estimated $Z_{i}$.

# Section S3 Accuracy of Eigenvalue Estimation of Synthetic Data

Figure S1 shows the MAE for the estimates of the top eight largest eigenvalues of $W$. The MAE values were calculated and are shown for the square root of the eigenvalues. In contrast to the variance estimation, the TS/OLS did not indicate uniformly higher MAE values. In plot (a), the shape of the curve for the REM method was irregular. The MAE value of the REM estimate first decreased, then increased, and finally reached a constant level. This was presumably a consequence of bias in the maximum-likelihood estimators. Although there are no definite criteria for the accuracy of the eigenvalues of protein conformational variability, the presented accuracy is sufficient for determining the essential eigenvalues and eigenvectors. For intra-ensemble variability $\Sigma$, the values of ME and MAE were low in general, probably owing to the larger effective sample sizes. The MAE values of the TS/OLS estimates were slightly higher than those of the other methods, as in the case of the variances.


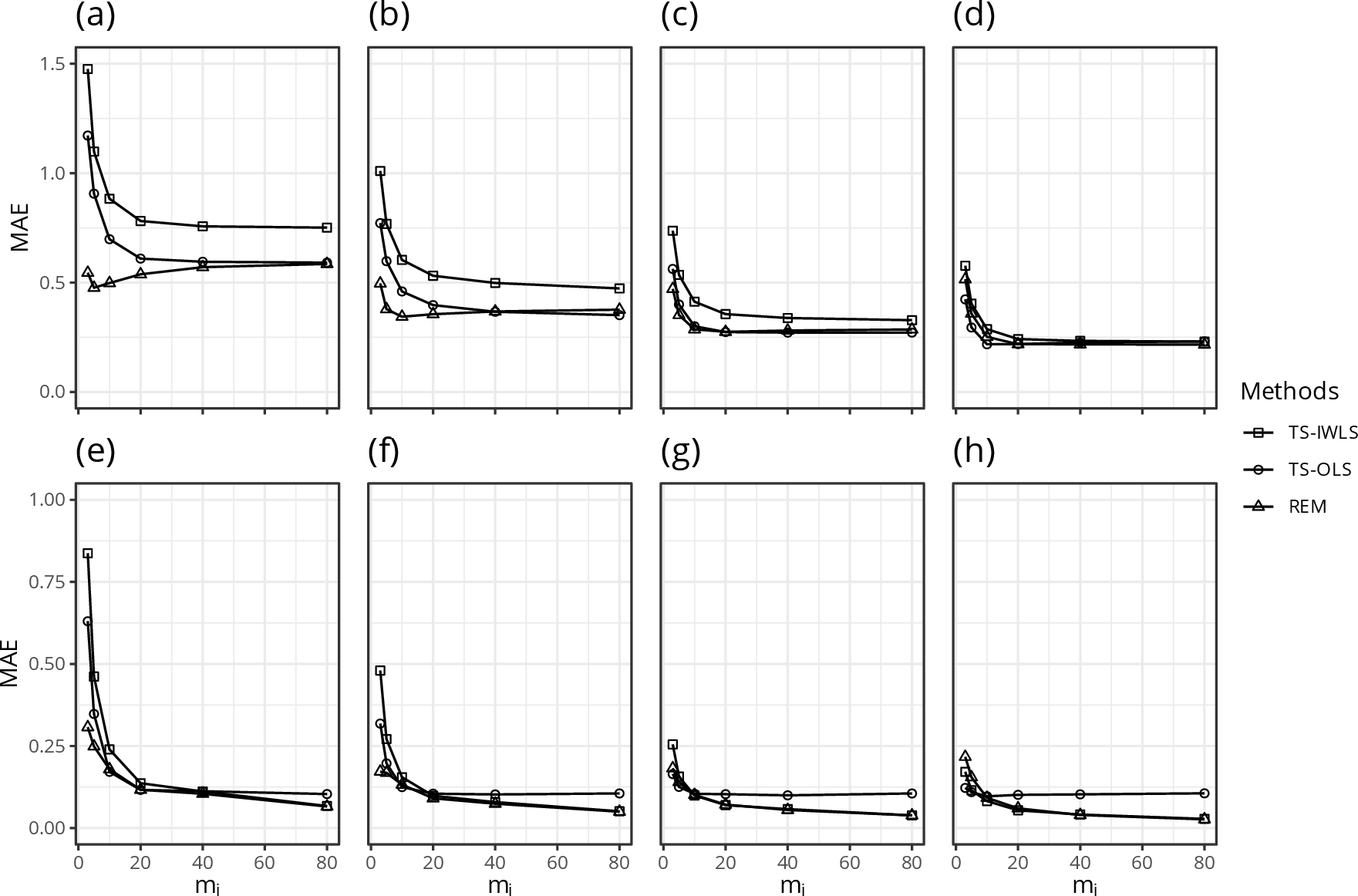


Figure S1: The mean absolute error (MAE) of the square root of the estimates for the top eight eigenvalues as functions of $m_{i}$. The upper four panes are the plots for $\hat{W}$ with $\nu=10$(a), 20(b), 40(c), and $80$(d). The lower panes are the respective plots for $\hat{\Sigma}$. The averages for the true square root for the top eight eigenvalues of $W$ and $\Sigma$ were 4.07 and 3.47, respectively. At low $m_{i}$ and $\nu$, the accuracy of the REM estimates was relatively higher than those of the other methods.

# Tables

Table S1. List of the structures of human CYP2 enzymes. Each code is composed of PDB ID (four letters) and chain ID (one capital letter).

| Enzyme | Codes |
| --- | --- |
| 2A6 | 1z10A 1z10B 1z10C 1z10D 1z11A 1z11B 1z11C 1z11D 2fduA 2fduB 2fduC 2fduD 2fdvA 2fdvB 2fdvC 2fdvD 2fdwA 2fdwB 2fdwC 2fdwD 2fdyA 2fdyB 2fdyC 2fdyD 3ebsA 3ebsB 3ebsC 3ebsD 3t3qA 3t3qB 3t3qC 3t3qD 3t3rA 3t3rB 3t3rC 3t3rD 4ejjA 4ejjB 4ejjC 4ejjD 4ruiA 4ruiB 4ruiC 4ruiD 4ruiE 4ruiF |
| 2B6 | 3ibdA 3qoaA 3qu8A 3qu8B 3qu8C 3qu8D 3qu8E 3qu8F 3ua5A 3ua5B 4i91A 4rqlA 4rqlB 4rrtA 4rrtB 4zv8A 5uapA 5uapB 5udaA 5udaB 5uecA 5ufgA 5wbgA 5wbgB 5wbgC 5wbgD 5wbgE 5wbgF |
| 2C8 | 1pq2A 1pq2B 2nnhA 2nnhB 2nniA 2nnjA 2vn0A |
| 2C9 | 1og2A 1og2B 1og5A 1og5B 1r9oA 4nz2A 4nz2B 5a5iA 5a5jA 5k7kA 5x23A 5x24A 5xxiA 6vltA 6vltB 6vltC 6vltD 6vltE 6vltF 6vltG 6vltH 7rl2A |
| 2D6 | 2f9qA 2f9qB 2f9qC 2f9qD 3qm4A 3qm4B 3tbgA 3tbgB 3tbgC 3tbgD 3tdaA 3tdaB 3tdaC 3tdaD 4wntA 4wntB 4wnuB 4wnuC 4wnuD 4wnvA 4wnvB 4wnvC 4wnvD 4wnwA 4wnwB 4xryA 4xryB 4xryD 4xrzA 4xrzB 4xrzC 4xrzD 5tftA 5tftB 5tftD 5tfuA 5tfuB 5tfuC 5tfuD 6csbA 6csbB 6csbC 6csbD 6csdA 6csdB |
| 2E1 | 3e4eA 3e4eB 3e6iA 3e6iB 3gphA 3gphB 3kohA 3kohB 3lc4A 3lc4B 3t3zA 3t3zB 3t3zC 3t3zD |

Table S2. List of the structures of class A $\beta$-lactamases. Each code is composed of PDB ID (four letters) and chain ID (one capital letter).

| Enzyme | Codes |
| --- | --- |
| BEL-1 | 5eoeA 5eooA 5ephD 5euaA |
| BPS-1 | 3w4oA 3w4pA |
| cTEM-19m | 4qy5A 4qy6A 4r4rA 4r4sA |
| CTX-M-14 | 1yltA 1ylyA 1ylzA 4xxrA 5toyA 5tweA 6bt6A 6md8A 6miaA 6mz1A 6mz1B 6mz2A 6oofA 6oojA 6ookA 6unbA 6v5eA 6v6pA 6v7hA 6v7tA 6v7tB |
| CumA | 1hzoA 1hzoB |
| GES-1 | 2qpnA 2qpnB 4gogA 4gogB |
| KPC-2 | 3rxwA 3rxxA 4zbeA 5eecA 5eecB 5uj3A 5uj4A 5ul8A 6b1fA 6b1fB 6b1hA 6b1hB 6b1jA 6b1jB 6b1wA 6b1wB 6b1xA 6b1xB 6b1yA 6b1yB 6d15A 6d16A 6d17A 6d18A 6d19A 6j8qA 6j8qB 6j8qC 6jn3A 6jn3B 6jn3C 6jn4A 6jn4B 6jn4D 6jn5A 6jn5C |
| L2 | 1n4oA 1n4oB 1o7eA 1o7eB 5ne1A 5ne1B 5ne2A 5ne2B 6qw7A 6qw7B |
| NmcA | 1bueA 1bulA |
| PenL | 5gl9A 5glaA 5glaB 5glbA 6afmA 6afnA 6afoA 6afoB 6afpA 6afpB |
| PSE-4 | 1g68A 1g6aA |
| SHV-1 | 1ongA 1q2pA 1rcjA 1shvA 1tdgA 1tdlA 1vm1A 2a3uA 2a49A 2g2uA 2g2wA 2h0tA 2h0yA 2h10A 2h5sA 2zd8A 3c4oA 3c4pA 3d4fA 3mkeA 3mkfA 3mxrA 3mxsA 3n4iA 3ophA |
| SME-1 | 1dy6A 1dy6B |
| TEM-1 | 1axbA 1bt5A 1btlA 1ck3A 1ermA 1eroA 1erqA 1esuA 1fqgA 1jtdA 1jtgA 1jvjA 1jwpA 1jwvA 1jwzA 1lhyA 1li0A 1li9A 1m40A 1nxyA 1ny0A 1nymA 1nyyA 1pzoA 1pzpA |

# Figures


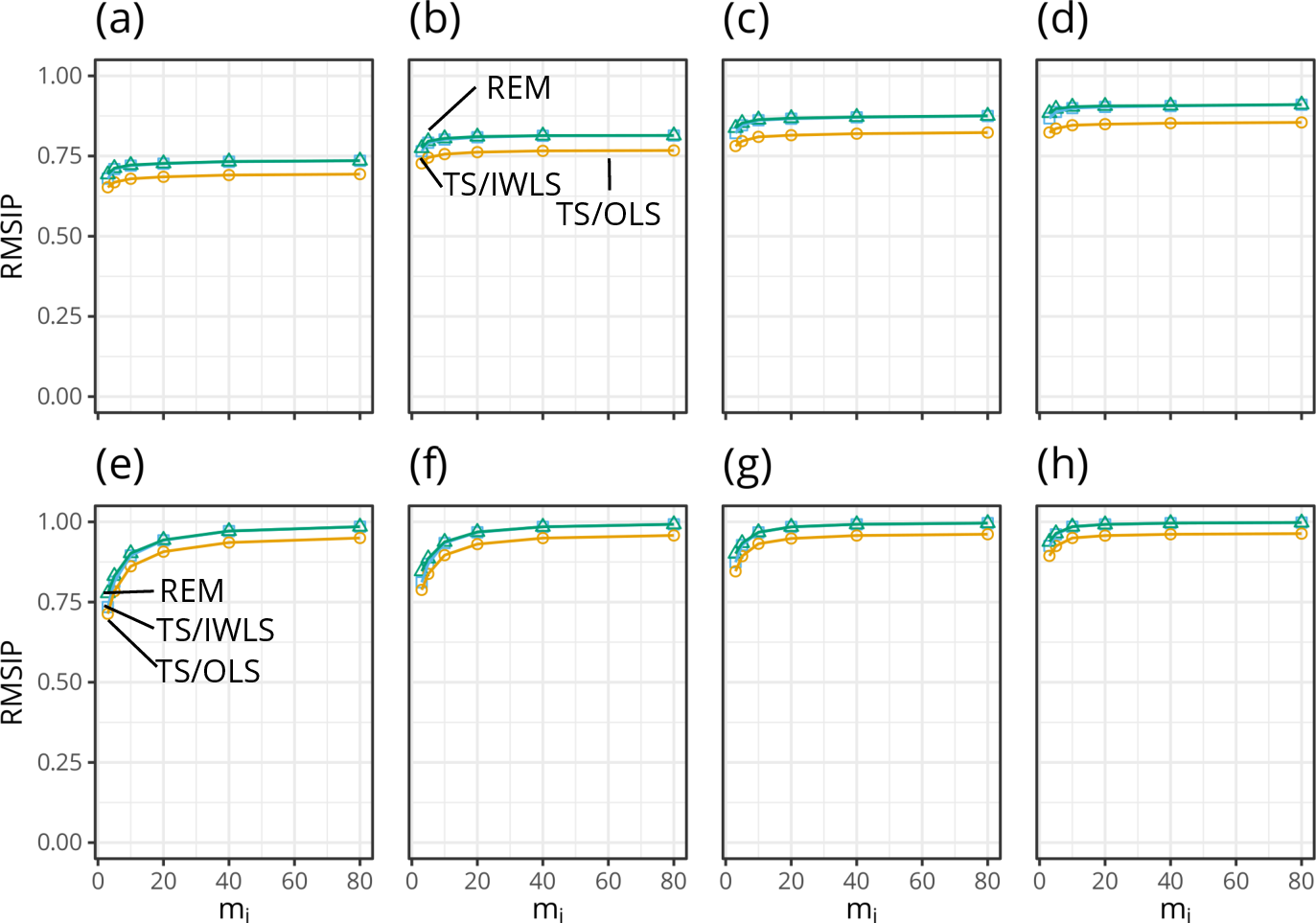


Figure S2: The root mean squared inner product (RSMIP) values of the top eight eigenvectors as a function of $m_{i}$. The upper four panes are the plots for $\hat{W}$ with $\nu=10$(a), 20(b), 40(c), and $80$(d). The lower panes are the respective plots for $\hat{\Sigma}$.


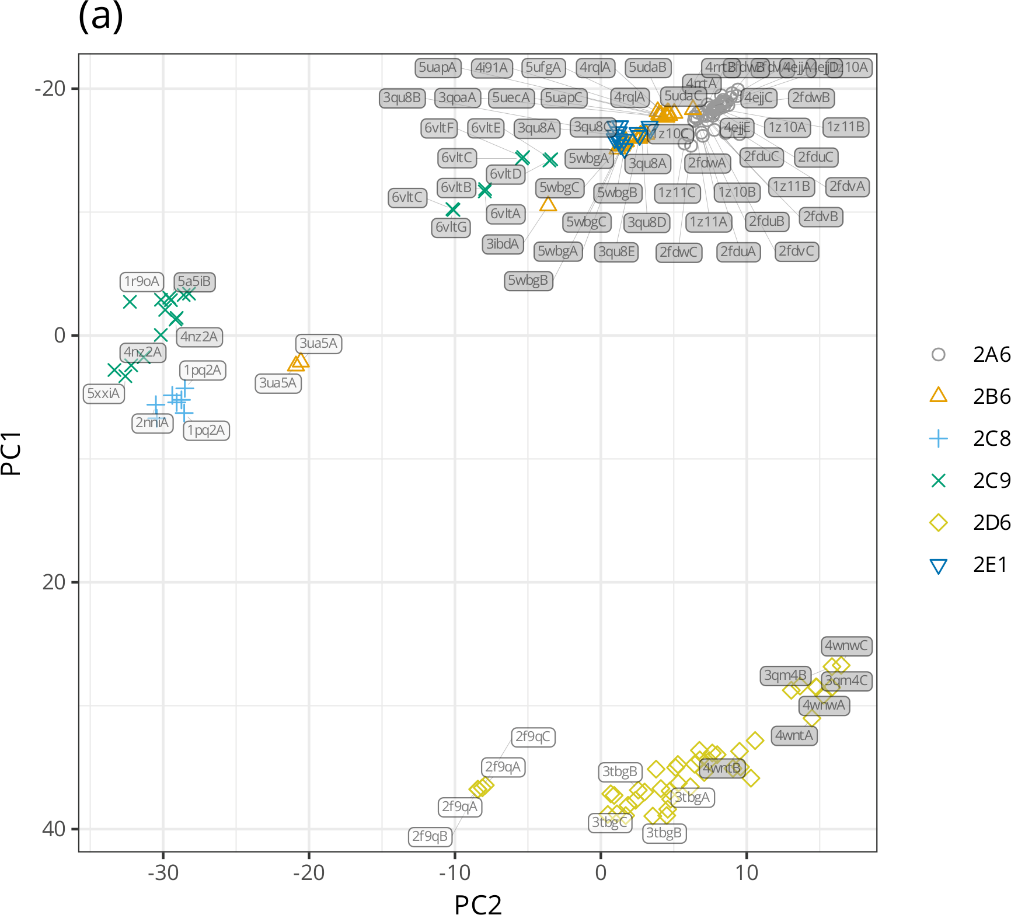

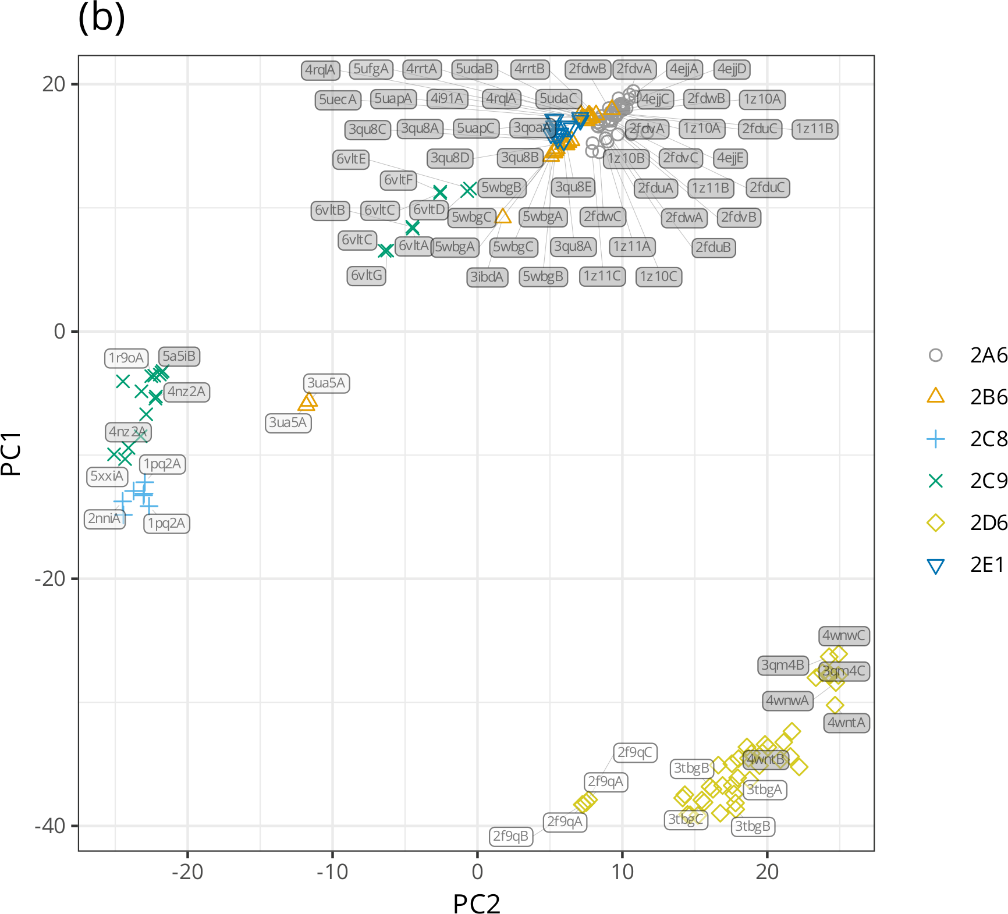


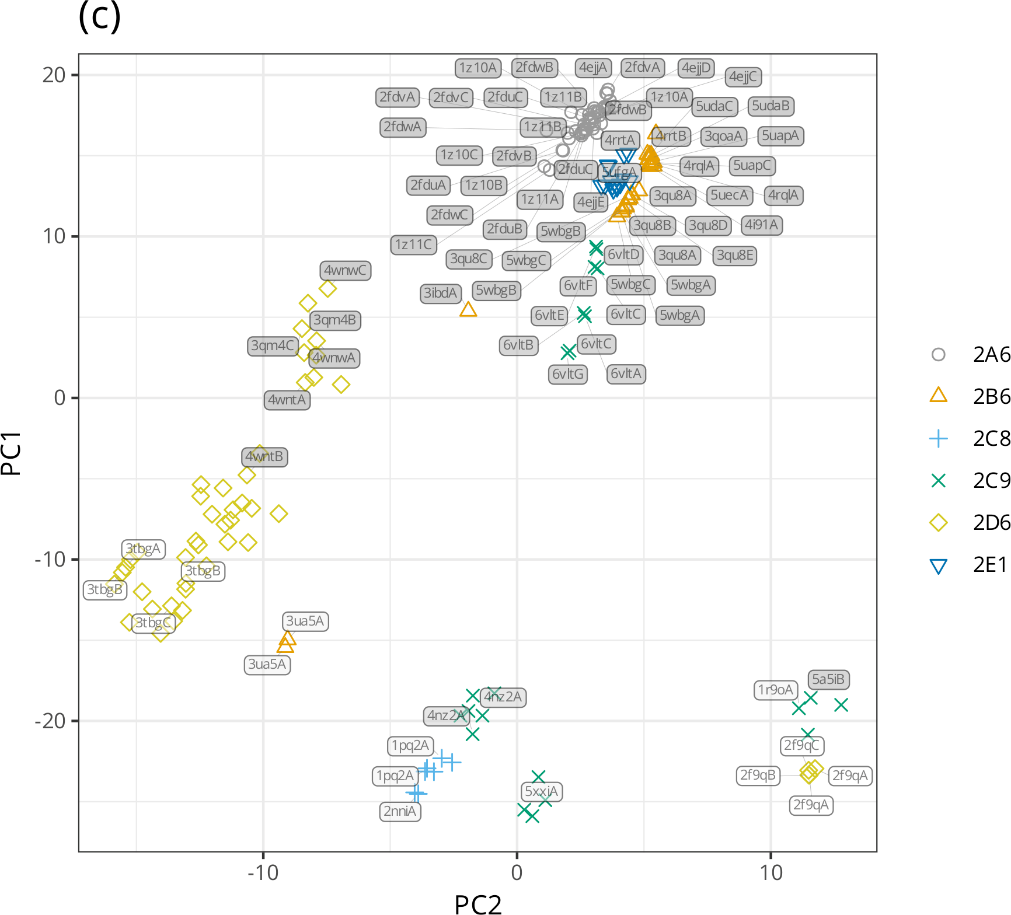


Figure S3: Projection of X-ray structures of CYP2 enzymes onto the PC2–PC1 plane of the covariance matrices: (a) PSE/IWLS $\hat{\Delta}$, (b) REM $\hat{W}$, and (c) REM $\hat{\Sigma}$. The label for each structure is composed of the PDB code followed by the chain ID. The gray tone of the labels is in five-grade, indicating the likelihood in the open conformation. Brighter labels indicate more likely to be in open.


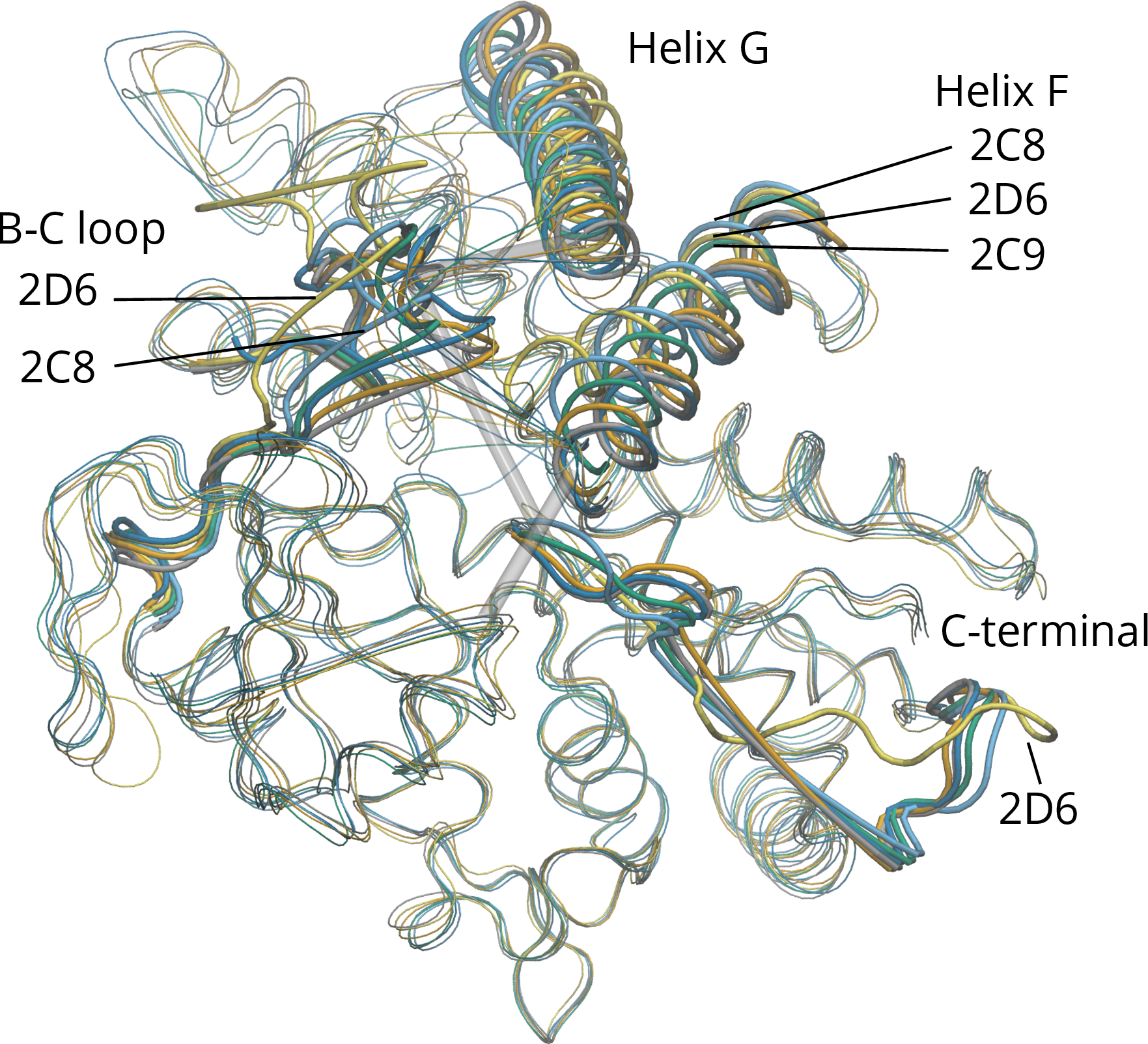


Figure S4: Estimated and superimposed average structures of the six CYP2 enzymes using TS/IWLS. The gray rods indicate dist1, dist2, and dist3. Regions that show large deviations in Figure 5 are indicated by thick tubes. Helix F of 2D6 (yellow), 2C8 (light blue), and 2C9 (green) inflated toward the upside, and the B–C loop of 2D6 and 2C8 largely inflated toward the upside. These two features were distinguished as the inter-ensemble variability, as involved in $\hat{\Delta}$ and $\hat{W}$, and appeared in each PC1 of the two covariance matrices.

# Section S4 Movies of the PC1 for CYP2 in the Cartesian Space

Movies S1 and S2 show the motions corresponding to PC1’s of $\hat{\Delta}$ and $\hat{\Sigma}$, respectively, in three-dimensional space. Specifically, each PC1 was projected onto the Cartesian coordinate space, interpolated between the maximum and minimum values in all conformations, and added to the estimated grand-average structure $\hat{M}$. In both movies, the open/closed motion of the F–G region (helices F and G connected by a loop) was remarkable. In addition, in the case of the PC1 of $\hat{\Delta}$, the B–C and C-terminal loops were greatly moved, coupled with the motion of the F–G region. The concerted motion in the PC1 of $\hat{\Delta}$ resulted in cavity stretching. On the other hand, in the case of PC1 of $\hat{\Sigma}$, there was no remarkable motion other than that of the F–G region that significantly changed the cleft size. Thus, the PC1 of REM $\hat{\Sigma}$ appeared to purely capture the open/closed motion of the cavity without being affected by the size distinction between the enzymes.

Movies S1 and S2 are provided as separate files.

# Section S5 An Application to Calmodulin

This is an example of the application to NMR ensembles alongside X-ray structures of a single protein, calmodulin. The protein possesses great flexibility at the linker helix connecting N- and C- domains, and exhibits large conformational changes upon binding to Ca^2+^ and target proteins[9].

As stated in the main text, it is widely considered that extreme structural changes make structural superposition meaningless. However, in the following dataset of calmodulin structures, the rearrangement of the two domains was captured in the covariance matrices which were estimated using heteroscedastic REM and IWLS.

### Data and Methods

PDB files of the 23 NMR and 17 X-ray entries for UniProt ID P0DP23 were downloaded. Incomplete chains and residues were omitted. Finally, a dataset composed of 491 (NMR 422, X-ray 69) conformations of 137 C𝛼 atoms was obtained. Covariance estimation and eigen decomposition were performed as described for CYP2 and ABL. The structures were grouped by the following rules:

1. The NMR ensemble of a PDB entry was considered as a single group by itself.
2. The X-ray structures in PDB codes of the same first three letters were considered as the same group. This rule was intended to group a series of similar experiments conducted in a research project.
3. A group did not contain different variants.
4. All chains in an entry were assigned to the same group.

Entries 6b8l, 6b8m, 6b8n, and 6b8p were exceptions to Rule 2 because they each contained four structures and were each considered as a group.

The data are available on the GitHub repository.

### Results in Addition to Figure 9

The coordinates of the structures in the highest three components, PC1, PC2, and PC3, of the estimated covariance matrices were transformed into the corresponding Cartesian coordinates, and the magnitudes are plotted per residue in Figure S5.

The difference between the heteroscedastic (a,b,c) and homoscedastic (d,e) methods is clear. The results of the heteroscedastic methods imply large structural changes, which are observed in the superimposed structures (Figure 9(a)). On the other hand, the displacements corresponding to the PCs of the homoscedastic methods (Figure S5(d,e)) appear to be the results of unweighted superposition, which copes with the two domains evenly (Figure 9(b)).

In Figure S5, the PC coordinates were almost similar between SPE/IWLS $\hat{\Delta}$ and REM $\hat{W}$, while in REM, the twin peaks of $\hat{W}$ PC3 appeared in the C-domain. The peaks seemed to arise from the X-ray structures, as the corresponding peaks appeared more clearly in the PC2 of SPE/IWLS $\hat{\Delta}$ as well as that of REM $\hat{W}$, when the covariance matrices were estimated using X-ray data alone.

To determine why the structures in Figure 9(a) are solely superimposed on the N-domain, several experiments were conducted. It appeared that the REM method converged either to N-domain superimposed or to C-domain superimposed structures presumably depending on which domain contained more Cα atoms. Twenty superpositions with varying numbers of Cα atoms, ignoring zero to nineteen N-terminal residues, are shown for REM and TS/IWLS in Figures S6(a) and S6(b), respectively. The REM method best superimposed the N-domain if less than eight N-terminal residues were excluded from its calculation, and best superimposed the C-domain otherwise. On the other hand, in the results of the TS/IWLS method, the superimposed part gradually transitioned from the N-terminal to the C-terminal as more residues at the N-terminal were excluded.

Finally, it would be worthwhile to discuss the problem of local minima and related things. Figure S7 shows the results of REM-superpositions starting from different initial superpositions. In the current implementation of REM, each structure is initially superimposed onto the first structure before entering the REM iteration. Figures 9 and 7(a) show the results starting from this initial position. The REM method converged to a point with $-2\log L_{o}=7.8291\times{10}^{5}$. When initial superposition was carried out using only the N-terminal half (residues 1-77), the method converged to the same superposition (Figure S7(b)). When initial superposition was carried out using only the C-terminal half (residues 78-137), the method converged to a point with $-2\log L_{o}=8.6977\times{10}^{5}$ (Figure S7(c)). Furthermore, when superimposed structures (a) were subject to one step of the REM procedure imposing very low weights ($1\times{10}^{-30}$) to the N-terminal half and then used as an initial superposition, the method converged to a point with $-2\log L_{o}=8.7264\times{10}^{5}$ (Figure S7(d)). The three points were possibly distinct local minima. Accurately, some or all the points may not be true local minima but may be points where the algorithm or its implementation was stuck [10, p.674].


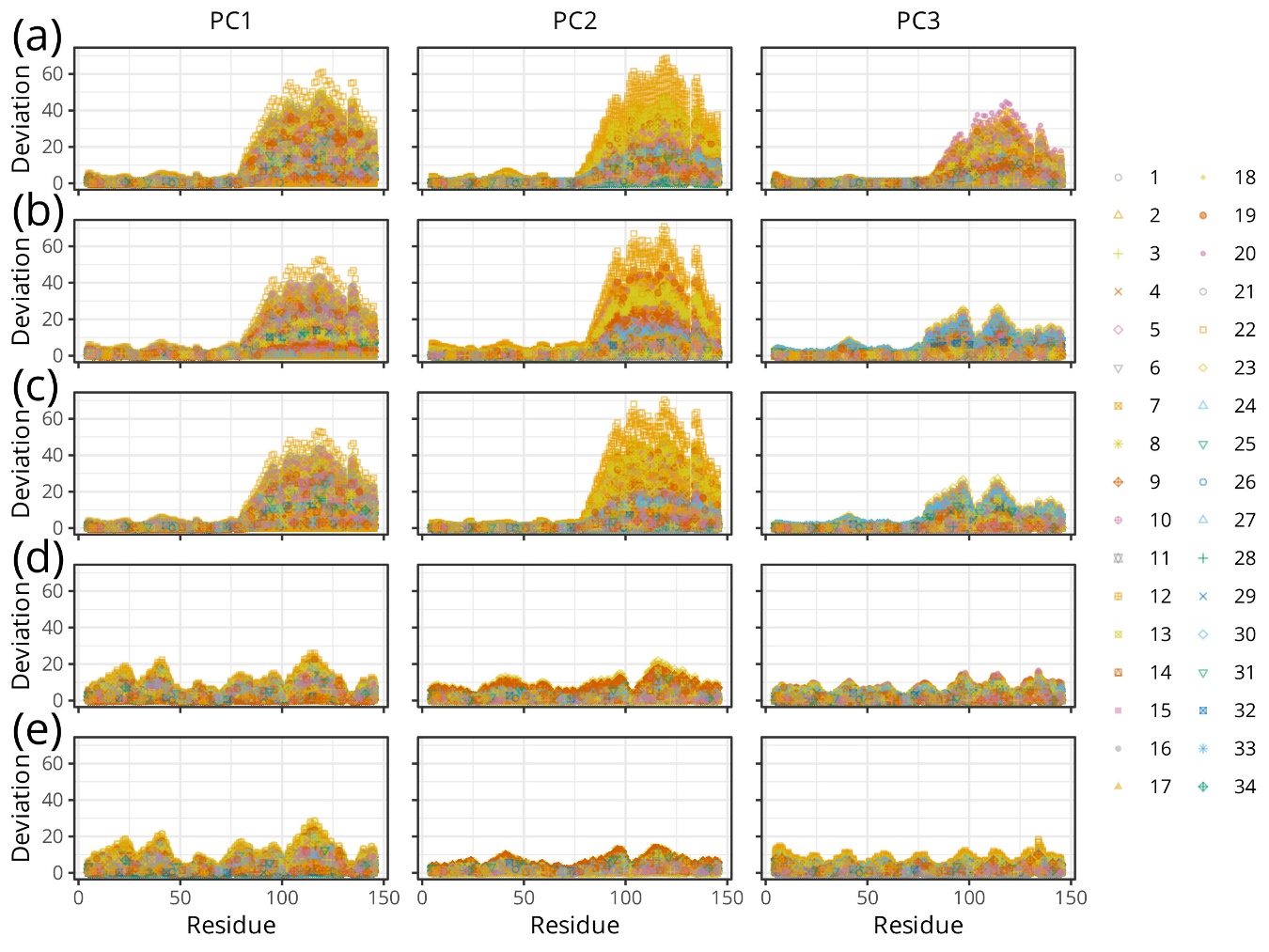


*Figure S5. Displacement of the* *C*$\alpha$ *atoms of each structure. The coordinates in the first three PCs of (a) PSE/IWLS* $\hat{\Delta}$*, (b) TS/IWLS* $\hat{W}$*, (c) REM* $\hat{W}$*, (d) PSE/OLS* $\hat{\Delta}$*, and (e) TS/OLS* $\hat{W}$ *were transformed to the deviations in the Cartesian coordinates (Å) and plotted per residue. The groups of numbers* $\leq$ *23 are the NMR ensembles, and the numbers* $>$ *34 are the X-ray ensembles. In (b), results for a group were not shown, because the first stage IWLS calculation for that group failed.* $\hat{W}$ *was calculated without the mean structure of the group.*


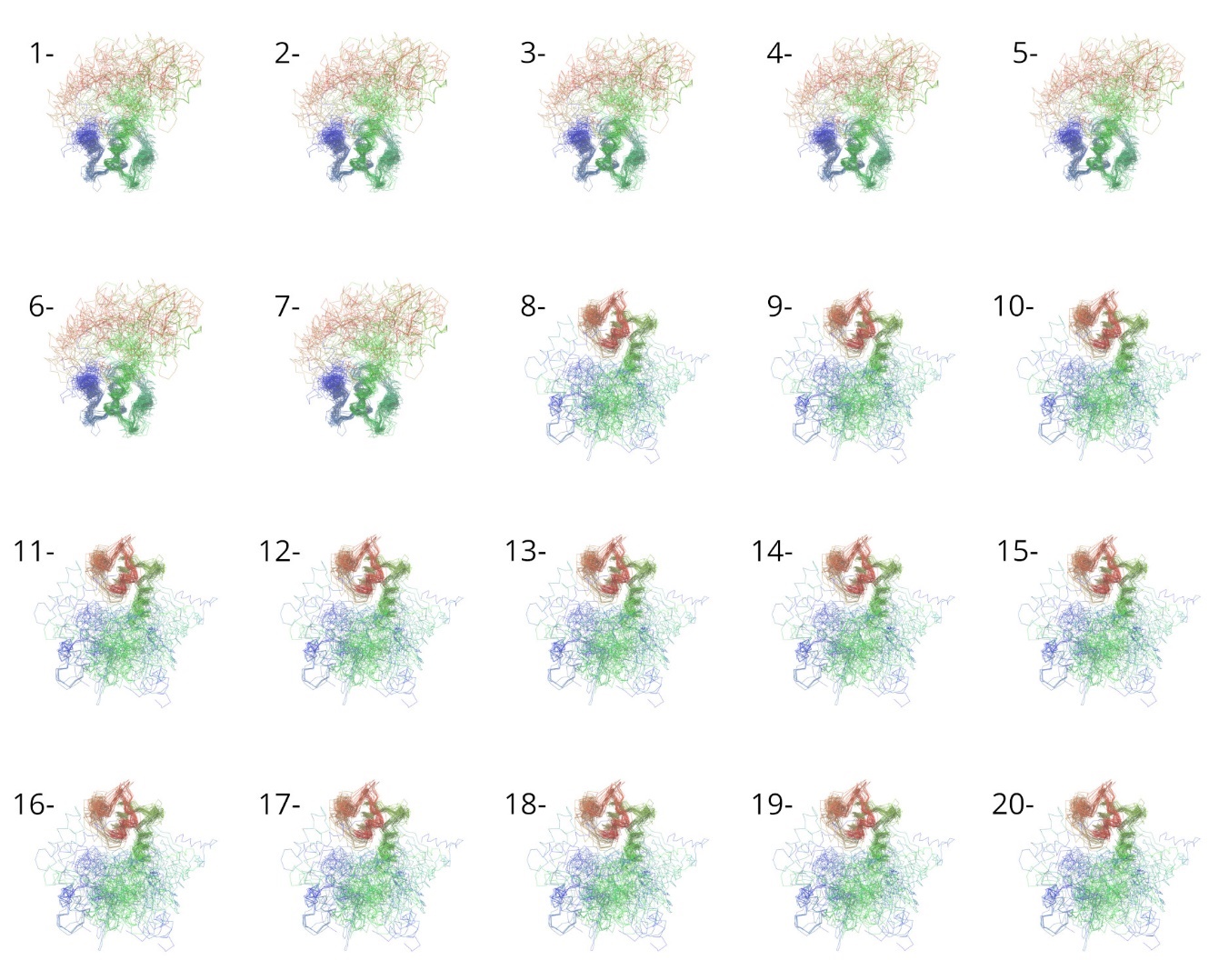


*Figure S6 (a). The REM-superpositions of calmodulin structures using varying numbers of residues. The traces of the C*$\alpha$ *atoms of the mean group structures are shown. Each trace is colored in a blue-green-red scale from the N- to the C-terminal. Each superposition was carried out using the residues starting from the labeled number to the C-terminal. For example, superposition 18- shows the structures superimposed only using residues 18-137.*


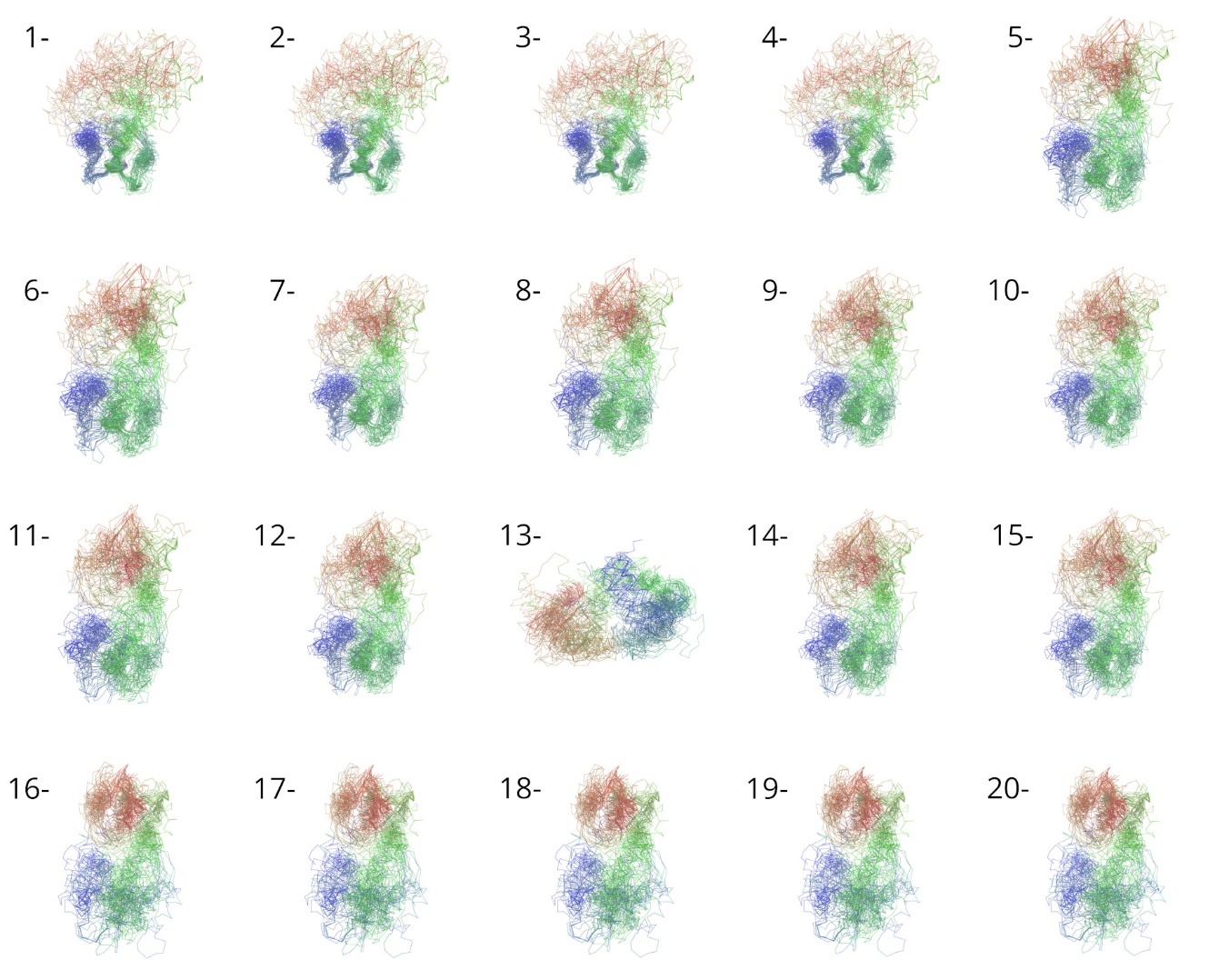


*Figure S6 (b). The TS/IWLS-superpositions of calmodulin structures using varying numbers of residues. The traces of the C*$\alpha$ *atoms of the mean group structures are shown. Each trace is colored in a blue-green-red scale from the N- to the C-terminal. Each superposition was carried out using the residues starting from the labeled number to the C-terminal. For example, superposition 18- shows the structures superimposed only using residues 18-137.*


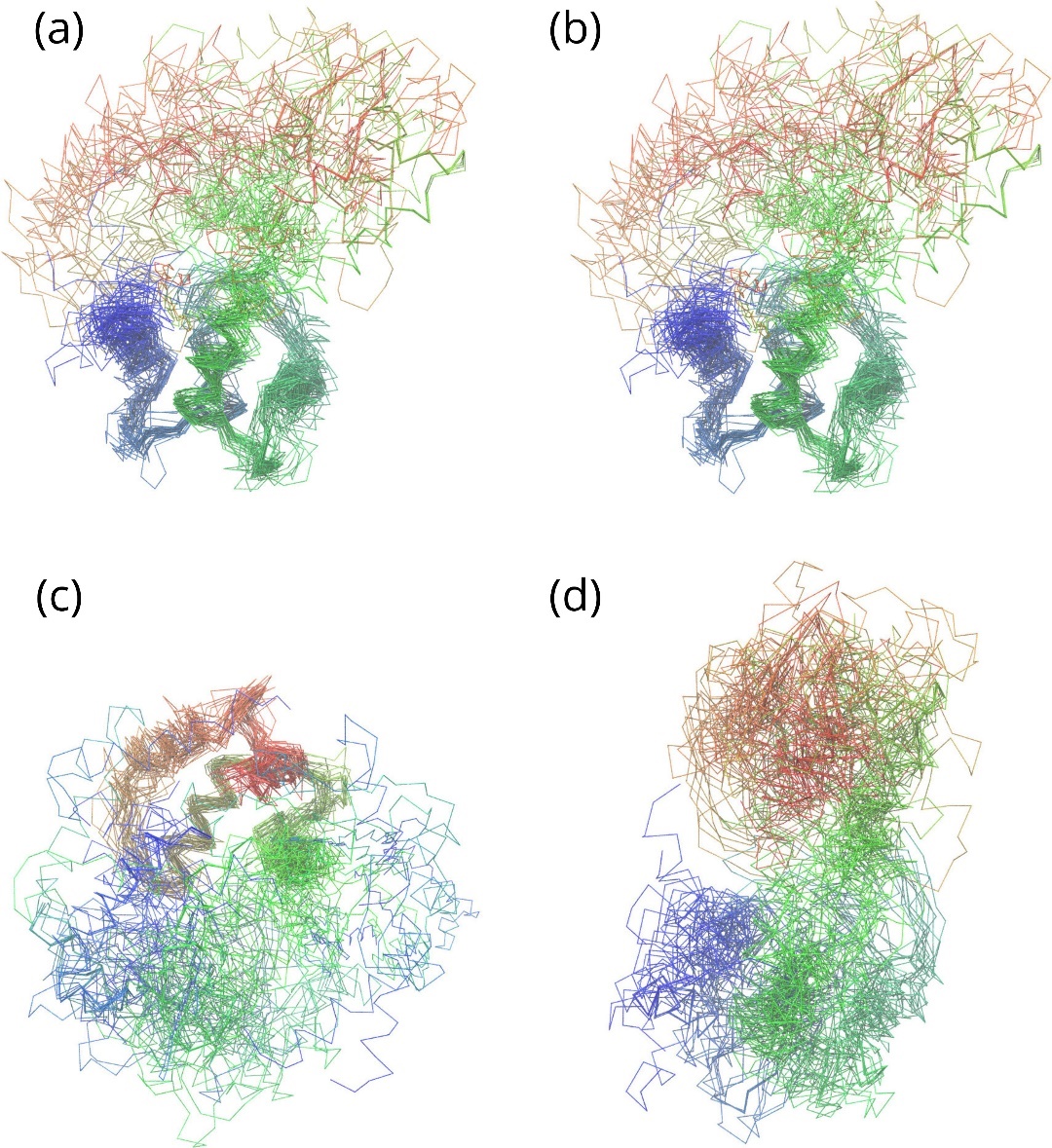


*Figure S7. The results of REM-superposition starting from different initial superpositions. The C*$\alpha$ *traces of the mean group structures of calmodulin are shown. Each trace is colored in a blue-green-red scale from the N- to the C-terminal. (a) Each structure was initially superimposed onto the first structure. (b) Each structure was initially superimposed onto the first structure using only the N-terminal half (residues 1-77). (c) Each structure was initially superimposed onto the first structure using only the C-terminal half (residues 78-137). (d) The superimposed structures (a) were subject to one step of the REM procedure imposing very low weights to N-terminal half (residues 1-77) and then used as an initial superposition.*

# References

1. Theobald DL, Wuttke DS. Empirical Bayes hierarchical models for regularizing maximum likelihood estimation in the matrix Gaussian Procrustes problem. Proc. Natl. Acad. Sci. U.S.A. 2006; 103:18521–18527.

2. Chen J, Shao J. Iterative Weighted Least Squares Estimators. Ann. Statist. 1993; 21: 1071–1092.

3. Hooper PM. Iterative Weighted Least Squares Estimation in Heteroscedastic Linear Models. J. Am. Stat. Assoc. 1993; 88:179–184.

4. Carroll RJ, Ruppert D. Transformation and Weighting in Regression. New York: Chapman & Hall, 1988, page 14.

5. Kabsch W. A solution for the best rotation to relate two sets of vectors. Acta Cryst. A 1976; 32:922–923.

6. Laird NM, Ware JH. Random-effects models for longitudinal data. Biometrics 1982; 38:963–974.

7. McLachlan GJ, Krishnan T. The EM algorithm and extensions. Oxfordshire: Taylor and Francis, 2008.

8. Cossio-Pérez R, Palma J, Pierdominici-Sottile G. Consistent Principal Component Modes from Molecular Dynamics Simulations of Proteins. J. Chem. Inf. Model. 2017; 57:826–834.

9. Zhang M, Abrams C, Wang L et al. Structural Basis for Calmodulin as a Dynamic Calcium Sensor. Structure 2012; 20:911-923.

10. Demidenko E. Mixed models: Theory and applications with R. New Jersey: John Wiley & Sons, 2013.
